# *Andrographis paniculata* transcriptome provides molecular insights into tissue-specific accumulation of medicinal diterpenes

**DOI:** 10.1186/s12864-015-1864-y

**Published:** 2015-09-02

**Authors:** Anchal Garg, Lalit Agrawal, Rajesh Chandra Misra, Shubha Sharma, Sumit Ghosh

**Affiliations:** Biotechnology Division, Council of Scientific and Industrial Research-Central Institute of Medicinal and Aromatic Plants, Lucknow, 226015 India; Council of Scientific and Industrial Research-National Botanical Research Institute, Lucknow, 226001 India

**Keywords:** *Andrographis paniculata*, *ent*-labdane-related diterpene, Medicinal compound, Specialized metabolism, Transcriptome

## Abstract

**Background:**

Kalmegh (*Andrographis paniculata*) has been widely exploited in traditional medicine for the treatment of infectious diseases and health disorders. *Ent*-labdane-related diterpene (*ent*-LRD) specialized (i.e., secondary) metabolites of kalmegh such as andrographolide, neoandrographolide and 14-deoxy-11,12-didehydroandrographolide, are known for variety of pharmacological activities. However, due to the lack of genomic and transcriptomic information, underlying molecular basis of *ent*-LRDs biosynthesis has remained largely unknown. To identify candidate genes of the *ent*-LRD biosynthetic pathway, we performed comparative transcriptome analysis using leaf and root tissues that differentially accumulate *ent*-LRDs.

**Results:**

*De novo* assembly of Illumina HiSeq2000 platform-generated paired-end sequencing reads resulted into 69,011 leaf and 64,244 root transcripts which were assembled into a total of 84,628 unique transcripts. Annotation of these transcripts to the Uniprot, Kyoto Encyclopedia of Genes and Genomes (KEGG) and Carbohydrate-Active Enzymes (CAZy) databases identified candidate transcripts of the *ent*-LRD biosynthetic pathway. These included transcripts that encode enzymes of the plastidial 2C-methyl-D-erythritol-4-phosphate pathway which provides C5 isoprenoid precursors for the *ent*-LRDs biosynthesis, geranylgeranyl diphosphate synthase, class II diterpene synthase (diTPS), cytochrome P450 monooxygenase and glycosyltransferase. Three class II diTPSs (*ApCPS1*, *ApCPS2* and *ApCPS3*) that showed distinct tissue-specific expression profiles and are phylogenetically related to the dicotyledon *ent*-copalyl diphosphate synthases, are identified. *ApCPS1*, *ApCPS2* and *ApCPS3* encode for 832-, 817- and 797- amino acids proteins of 55–63 % identity, respectively. Spatio-temporal patterns of transcripts and *ent*-LRDs accumulation are consistent with the involvement of *ApCPS1* in general (i.e., primary) metabolism for the biosynthesis of phytohormone gibberellin, *ApCPS2* in leaf specialized *ent*-LRDs biosynthesis and *ApCPS3* in root diterpene biosynthesis. Moreover, simple sequence repeats (SSRs) that might assist in genotyping and developing specific chemotypes were identified in transcripts of the specialized metabolic pathways, including *ent*-LRDs.

**Conclusions:**

Comparative analysis of root and leaf transcriptomes disclosed novel genes of the *ent*-LRD biosynthetic pathway, including three class II diTPSs that showed discrete spatio-temporal expression patterns; thus, suggesting their participation into distinct diterpene metabolic pathways of kalmegh. Overall, these results will be useful in understanding molecular basis of the medicinal *ent*-LRDs biosynthesis and developing breeding strategies for improving their yields.

**Electronic supplementary material:**

The online version of this article (doi:10.1186/s12864-015-1864-y) contains supplementary material, which is available to authorized users.

## Background

Kalmegh [*Andrographis paniculata* (Burm.f.) Wall. ex Nees], an annual herbaceous plant of the Acanthaceae family, is cultivated in Southern and Southeastern Asia for its diverse medicinal utilities [1, 2]. During global flu epidemic in 1919, medicinal properties of kalmegh were effectively exploited to arrest spread of the contagious illness [3]. The genus *Andrographis* comprises of about 40 species, among these kalmegh is most popular as medicinal plant [4]. Although kalmegh has been widely used in traditional medicine in several Asian countries, Southern parts of India and Sri Lanka are considered as the centre of origin and diversity of the *Andrographis* species. In India, kalmegh is a predominant constituent in several ayurveda, unani, siddha and tribal medicine formulations for the treatment of infectious diseases and health disorders [5, 6]. *In vitro* and *in vivo* bioactivity studies, using plant extracts as well as isolated compounds, revealed the utilities of kalmegh as hepatoprotective, anti-inflammatory, anticarcinogenic, anti-microbial, immunostimulatory, antioxidant and other health-promoting activities [2, 7–9]. KalmCold®, a clinically tested phytochemical composition of kalmegh, has been proven to be effective for the treatment of upper respiratory tract infection [10].

**Fig. 1:**
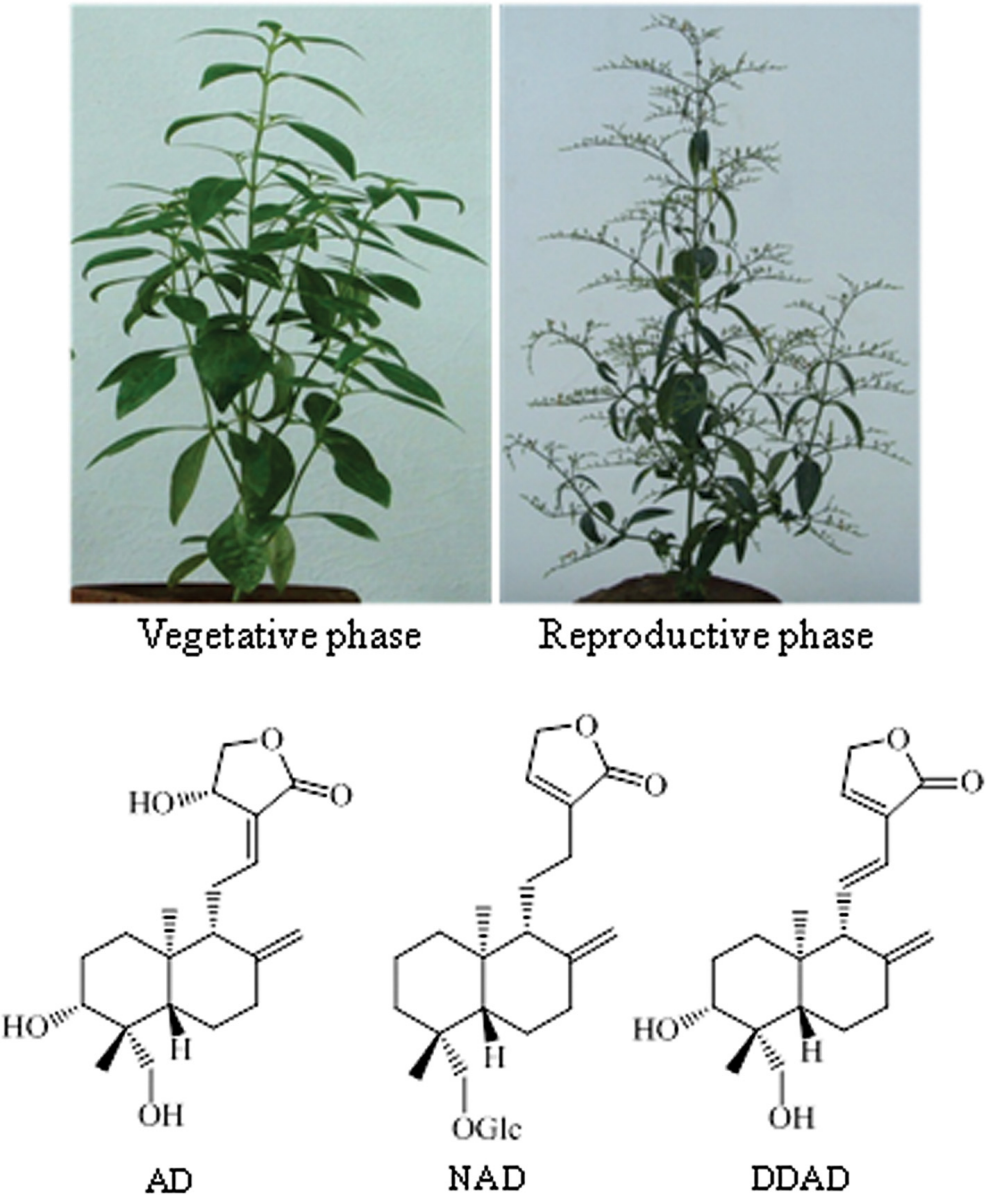
The medicinal plant kalmegh and major *ent*-LRDs that accumulate in leaves. AD, andrographolide; NAD, neoandrographolide; DDAD, 14-deoxy-11,12-didehydroandrographolide

Several bioactive specialized metabolites such as *ent*-labdane-related diterpenes (*ent*-LRDs), phenylpropanoids, flavonoids and xanthones were isolated from kalmegh [11, 12]. However, *ent*-LRDs that accumulate in leaves such as andrographolide (AD), neoandrographolide (NAD) and 14-deoxy-11,12-didehydroandrographolide (DDAD) are considered as main bioactive constituents of kalmegh [7, 13–17] (Fig. 1). Among these *ent*-LRDs, andrographolide, the bitter principle of kalmegh, is most abundant and has been extensively studied for pharmacological activities such as immunostimulatory, anti-inflammatory and anticarcinogenic activities [18–22]. Andrographolide has been shown to inhibit proliferation of cancer cells by mitotic arrest and by activation of the intrinsic apoptotic pathway [21]. Andrographolide has also been shown to protect against cigarette smoke-induced oxidative lung injury via augmentation of the activities of anti-oxidative enzymes [8]. The anti-inflammatory activity of andrographolide was attributed to inhibition of the nuclear factor (NF)-kB pathway [18, 20]. Moreover, andrographolide was suggested to be effective in reducing chronic stress-triggered pathologies by regulating corticosterone and cytokine homeostasis [23].

The low yield of bioactive principles in kalmegh has led to increased market rate. Although, good-quality dried leaves of kalmegh could be sold for US$5/kg, purified compounds and their derivatives may cost as much as US$100,000/kg from specialist chemical suppliers [7]. Understanding the molecular basis of specialized metabolite biosynthetic pathways and their regulation shall be effective in increasing yield as well as designing specific chemotypes following biotechnological and molecular breeding approaches [24, 25]. However, the genes/enzymes involved in the biosynthesis of medicinally active specialized metabolites of kalmegh are yet to be identified and functionally characterized. The lack of transcriptomic and genomic resources is a hindrance for understanding the specialized metabolite biosynthetic pathways of kalmegh. Only 41 Expressed Sequence Tags (ESTs) and 60 nucleotide sequences of kalmegh are available in the National Center for Biotechnology Information (NCBI) GenBank database. In recent times, high throughput transcriptome sequencing using Illumina short-read sequencing platform has become a powerful approach to develop reference transcriptome for gene identification in non-model plants [26–31].

In the present study, to generate a reference transcriptome of kalmegh for the identification of genes of the specialized metabolic pathways, transcriptome sequencing and *de novo* assembly have been performed using tissues that either accumulate high level of *ent*-LRDs (leaf) or do not accumulate *ent*-LRDs (root). Comparative analysis of leaf and root transcriptomes revealed candidate genes for the biosynthesis of *ent*-LRDs. Three class II diterpene synthases (diTPSs) are identified. These class II diTPSs showed discrete spatio-temporal expression patterns; suggesting their participation into distinct diterpene metabolic pathways of kalmegh.

## Methods

### Plant materials and growth conditions

Kalmegh (Cv. CIM-Megha) seeds were collected from the National Gene Bank for Medicinal and Aromatic Plants (CSIR-CIMAP). Seeds were germinated in pre-sterilized soil and, at the second true leaf stage, seedlings were transplanted into earthen pots (15 cm height and internal diameter) with a mixture (2:1) of soil and vermicompost. Plants were grown in a glass house during the months of July-October at 26–28 °C under the natural light. Plants were watered daily with application of Hoagland’s solution once a week. Roots, stems and matured green leaves were obtained from two-month-old plants. Samples were also collected at germinating seeds (GS) and seedlings at cotyledonary leaf stage (CLS). All samples were washed with RO water, frozen immediately in liquid nitrogen and stored at −80 °C.

### RNA isolation and cDNA library preparation

Total RNA was isolated using TRIzol reagent according to the manufacturer’s protocol (Invitrogen). cDNA library preparation was performed according to the Illumina TruSeq RNA library protocol outlined in “TruSeq RNA Sample Preparation Guide” (Part # 15008136; Rev. A; Nov 2010). In brief, mRNAs were isolated from 1.0 μg total RNA following Poly-A RNA purification method. Further, purified mRNAs were fragmented with divalent cations at 94 °C for 4 min and reverse-transcribed with Superscript III Reverse transcriptase by using random hexamers. Second strand cDNAs were produced in the presence of DNA Polymerase I and RNaseH, and cDNAs were cleaned up using Agencourt Ampure XP SPRI beads (Beckman Coulter). Following end repair and addition of A base, Illumina Adapters were ligated to the cDNAs and SPRI cleanup was performed. Amplification of the cDNA library was carried out following eight cycles of PCR for the enrichment of adapter-ligated fragments. The cDNA library was quantified using Nanodrop and validated for quality by running an aliquot on High Sensitivity Bioanalyzer Chip (Agilent). The libraries showed peak spread over a range of 250–700 bp with the effective sequencing insert size of 130–580 bp, excluding adaptor sequences.

### Transcriptome sequencing, raw data processing and *de novo* assembly

Paired-end (100 bp) sequencing of root and leaf cDNA libraries was carried out using Illumina HiSeq2000 platform. Sequence reads of the leaf and root libraries are deposited in the NCBIs Short Read Archive database under the accession number SRP044357. Raw reads were quality checked using SeqQC V2.2 tool (Genotypic Technology Pvt. Ltd.) to remove adapters and low quality bases. *De novo* assembly of processed reads was performed using Velvet-1.2.10 [32] for various hash length (k-mers). K-mer of 55 was found to be better than others considering various parameters like total number of contigs generated, maximum contig length, total contig length and less number of N’s. Further, Oases 0.2.08 [33] was performed using Velvet-1.2.10 assembly, for the generation of final transcripts for root and leaf tissues. These transcripts were clustered using CD-HIT [34] at 95 % sequence identity to generate a non-redundant reference transcriptome for kalmegh. The strategy for the transcriptome assembly is presented in Additional file 1: Figure S1.

### Transcriptome annotation and differential gene expression analysis

Transcripts were similarity searched (ncbi-BLAST-2.2.29) against the sequences of Acanthaceae family deposited at the Uniprot-viridiplantae database. In addition, annotation was also carried out with rice and Arabidopsis proteins available at the Uniprot database. Gene Ontology was predicted for the annotated transcripts using GO information from the Uniprot database. Metabolic pathway analysis was carried out using KAAS Server [35] following default parameters and using Arabidopsis and rice as the model organisms. Transcripts encoding predicted glycosyltransferase enzymes were identified following annotation against eukaryotic glycosyltransferase sequences collected from the Carbohydrate-Active Enzymes (CAZy) database [36]. Cytochrome P450 monoxygenase families were identified based on the sequence similarity to the cytochrome P450 monoxygenase sequences available at the Uniprot database. For the identification of transcription factor families, transcripts were annotated to the Plant Transcription Factor Database (http://plntfdb.bio.uni-potsdam.de/v3.0). For the digital gene expression analysis (DGE), processed reads were aligned to the assembled transcriptome after generating unigenes to obtain read counts using custom perl scripts (Genotypic Technology Pvt. Ltd.). Reads were first aligned using “Bowtie tool” [37] and “Awk scripting” was used to generate the read count profile from the output file (.sam) of Bowtie alignment. Differential gene expression analysis was carried out using DESeq software [38] considering root sample as control, as explained with a flow diagram in Additional file 1: Figure S2.

### Simple sequence repeats (SSRs) prediction

SSR prediction was carried out using MISA perl script (http://pgrc.ipk-gatersleben.de/misa/download/misa.pl). Transcripts were checked for mono-repeats occurring at least ten times, di-repeats occurring at least six times and tri/tetra/penta/hexa-repeats occurring at least five times within a sequence. SSR was classified as complex when two SSRs were identified within 100 bp distance of each other.

### Quantitative real time PCR

RNAs were isolated, treated with DNase I and purified using RNeasy Mini Kit (Qiagen) as described previously [39]. Three independent isolations consisting of at least three plants for each group were performed. RNA quality was monitored by analysing the A260/280 ratio and resolving on 1.2 % (*w/v*) agarose gel. Four microgram of total RNAs were reverse-transcribed using superscript II (Invitrogen) and processed for qRT-PCR analysis using 7900 HT Fast Real Time PCR (Applied Biosystems) as described previously [39]. Oligonucleotide primers used in qRT-PCR amplification are listed in Additional file 2: Table S1. Relative gene expression was determined based on the 2^-ΔΔCt^ method using *actin* (KJ494921) as endogenous control.

### High performance liquid chromatography

Fresh tissues from germinating seeds (GS), seedlings at cotyledonary leaf stage (CLS), roots, stems and leaves were harvested and quickly frozen in liquid nitrogen. Using liquid nitrogen, and with the help of a pestle and mortar, frozen tissue was ground into fine powder. Ground tissue (500 mg) was extracted twice with 5 ml of methanol, evaporated to dryness and finally dissolved into 2 ml of methanol. HPLC analysis was carried out using a HPLC-UV (Shimadzu LC-10A, Tokyo, Japan) system as described previously [40]. Stock solution (1 mg ml^−1^) of authentic standard andrographolide (Sigma) was prepared in methanol and used for standard curve preparation.

## Results and discussion

### Transcriptome sequencing, *de novo* assembly and quality assessment

**Fig. 2:**
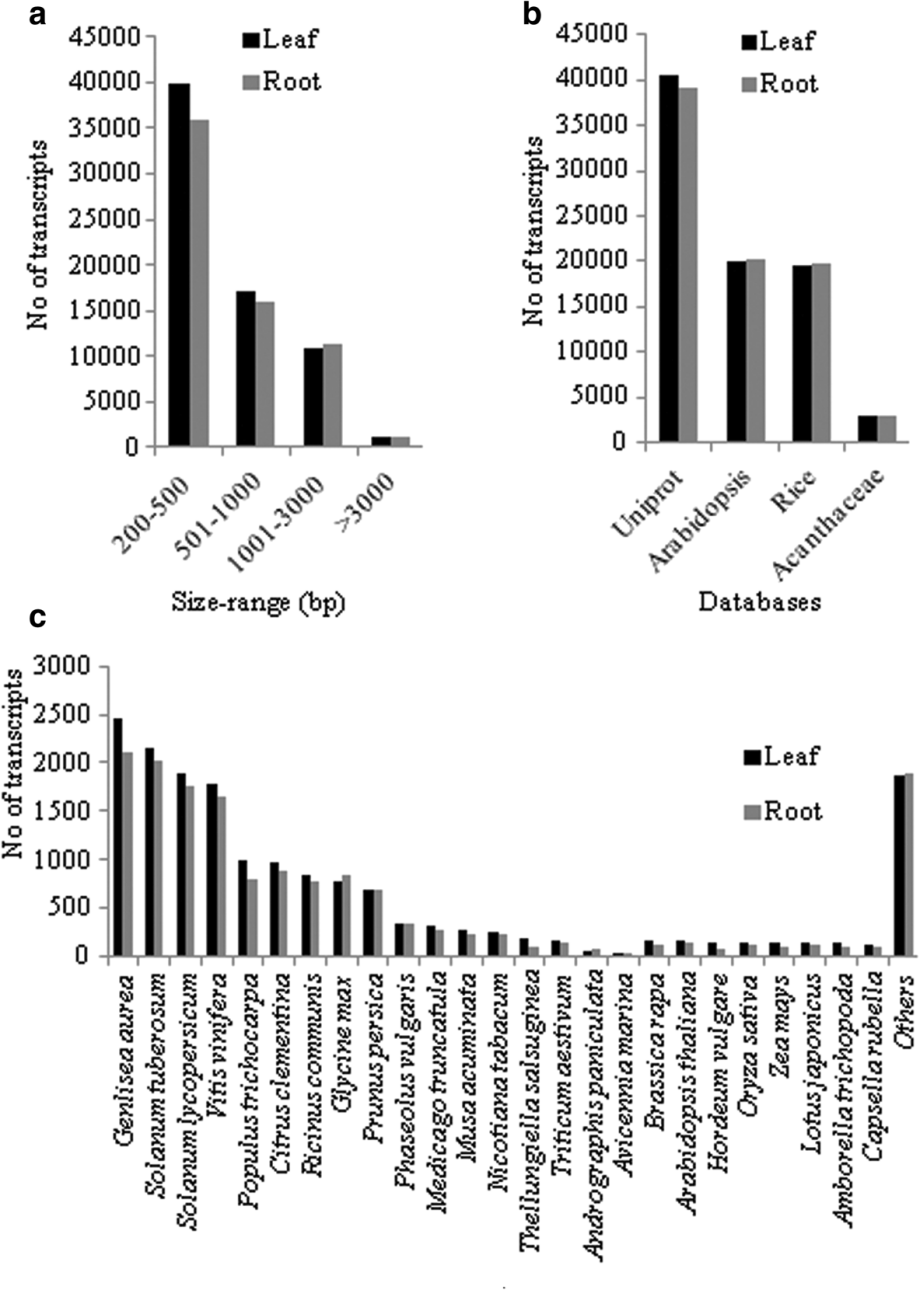
Size distribution and annotation of assembled transcripts. **a** Size distribution of transcripts. **b** Annotation of transcripts to different sequence databases was carried out on the basis of sequence similarity as determined by BLAST (version 2.2.29+) analysis. **c** Top-hit species distribution of transcripts showing ≥75 % sequence identity with annotated sequences of the Uniprot database

For organisms that lack reference genome, high throughput transcriptome sequencing using Illumina short-read sequencing platform combined with *de novo* transcriptome assembly has become a standard method in generating reference transcriptome with in-depth coverage [26–31]. Therefore, to identify candidate genes involved in the biosynthesis of medicinally active *ent*-LRDs, a reference transcriptome of kalmegh was generated following high-throughput sequencing of transcriptome using Illumina HiSeq2000 platform. As described in Materials and Methods, complementary DNA (cDNA) libraries were prepared from leaf and root tissues that accumulate contrasting levels of *ent*-LRDs [41] (Fig. 8b). Paired-end (100 bp) sequencing yielded 101.78 million and 78.48 million of raw reads for leaf and root, respectively. After processing of raw reads, 96.14 million and 74.85 million of high quality reads representing 9322.83 Mb and 7280.98 Mb of high quality bases were obtained for leaf and root, respectively. These high quality reads were utilized for *de novo* transcriptome assembly following Velvet_1.2.10 and Oases_0.2.08 software packages [32, 33]. The overall strategy of transcriptome assembly and analysis is presented in Additional file 1: Figure S1. By combining Velvet-1.2.10 and Oases_0.2.08 analyses, a total of 69,011 contigs with N50 of 926 bp and average length of 667 bp were generated for leaf transcriptome (Table 1). However, in case of root, a total of 64,244 contigs with N50 of 992 bp and average length of 692.5 bp were generated. The minimum length of leaf and root contigs was 200 bp. A large number of contigs (~55 %) were in the size range of 200–500 bp (Fig. 2a). On the other hand, ~25 % and ~15 % of contigs were in the size range of 501–1000 bp and 1001–3000 bp, respectively. These sequence data were in accordance to the transcriptomes reported for other plant species using Illumina short-read sequencing platform [27–30].

**Table 1:** Statistics for Illumina HiSeq2000 sequencing and *de novo* transcriptome assembly

Description	Leaf	Root
Total number of HQ reads	96,143,552	74,854,072
Total number of bases (Mb)	9563.22	7454.82
HQ bases (%)	97.48	97.66
Reads with non-ATGC characters (%)	0.32	0.33
GC content (%)	47.94	46.84
Reads assembled (%)	81.15	81.71
Total number of contigs	69,011	64,244
Average length (bp)	667	692.5
Total length (bp)	46,032,575	44,489,873
N50 (bp)	926	992

In order to evaluate quality of the assembled transcripts, identity and coverage of the sequences with the Uniprot protein database were determined following BlastX analysis (Additional file 3: Table S2 and Additional file 4: Table S3). The translation products of 87.25 % of the leaf and 86.80 % of the root annotated transcripts showed ≥50 % sequence identity with protein sequences deposited to the Uniprot database. Moreover, ≥70 % sequence coverage was recorded for 25.91 % (average length-1559.12 bp) of the leaf and 28.64 % (average length-1557.04 bp) of the root annotated transcripts, representing nearly full-length transcripts. Besides, ≥50 % sequence coverage was noticed for 39.06 % (average length-1360.97 bp) and 41.85 % (average length-1373.27 bp) of the leaf and root annotated transcripts, respectively. The average lengths of the transcripts with ˂50 % coverage were 508.64 and 516.22 bp for leaf and root, respectively. These parameters reflected the quality of the assembled transcripts.

### Annotation of the leaf and root transcriptomes

To assign putative function, transcripts were searched (BLAST version 2.2.29+) for homology to the annotated sequences in the Uniprot database, rice, Arabidopsis and Acanthaceae family (Fig. 2b; Additional file 3: Table S2 and Additional file 4: Table S3) and also processed for the Gene Ontology (GO) classifications (Additional file 1: Figure S3) and Kyoto Encyclopedia of Genes and Genomes (KEGG) pathway analysis (Additional file 5: Table S4 and Additional file 6: Table S5). Functional annotation was assigned for ~60 % of the transcripts based on similarity to the annotated sequences in the Uniprot database. However, ~30 % of the transcripts were annotated to the rice and Arabidopsis sequences. Because of limited transcript information on Acanthaceae family species, only ~4 % of the transcripts showed similarity with the annotated sequences of the Acanthaceae family species. BlastX search to the Uniprot database also provided information on to the species distribution of the annotated transcripts. When transcripts with ≥75 % of sequence identity to the annotated sequences in the Uniprot database were classified based on top-hit species distribution, *Genlisea aurea* represented top-hit species with similarity to 2446 leaf and 2114 root transcripts (Fig. 2c). This was in accordance with the fact that both *G. aurea* and *A. paniculata* belong to the order Lamiales. Only few transcripts showed similarity to the Acanthaceae family species such as *A. paniculata* and *Avicennia marina*. This reflected the lack of transcripts sequences of the Acanthaceae family species in the database.

**Fig. 3:**
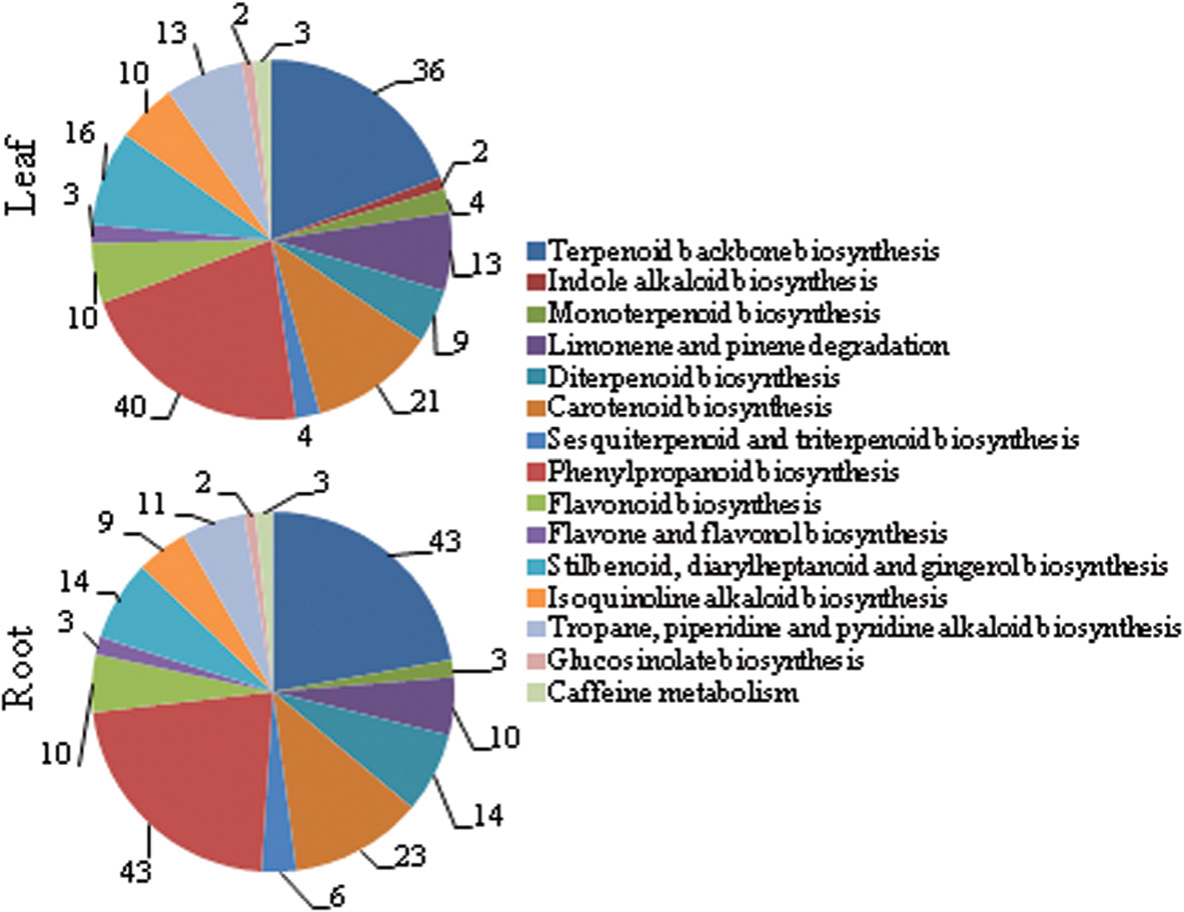
Annotation of transcripts to different specialized metabolic pathways based on the KEGG database

KEGG pathways were annotated to 4044 leaf and 3965 root transcripts (Additional file 5: Table S4 and Additional file 6: Table S5). In addition to several general (i.e., primary) metabolic pathways, transcriptome also represented various specialized (i.e., secondary) metabolic pathways such as terpenoids and phenylpropanoids (Fig. 3). Specialized metabolites of these classes were previously isolated from kalmegh, however, their biosynthetic pathway genes were not identified [11, 12, 42]. Functional analysis of the candidate genes of the specialized metabolic pathways shall be useful to understand the molecular and biochemical basis for the accumulation of medicinally active specialized metabolites in kalmegh.

### Combined assembly of leaf and root transcriptomes and identification of the differentially expressed transcripts

**Fig. 4:**
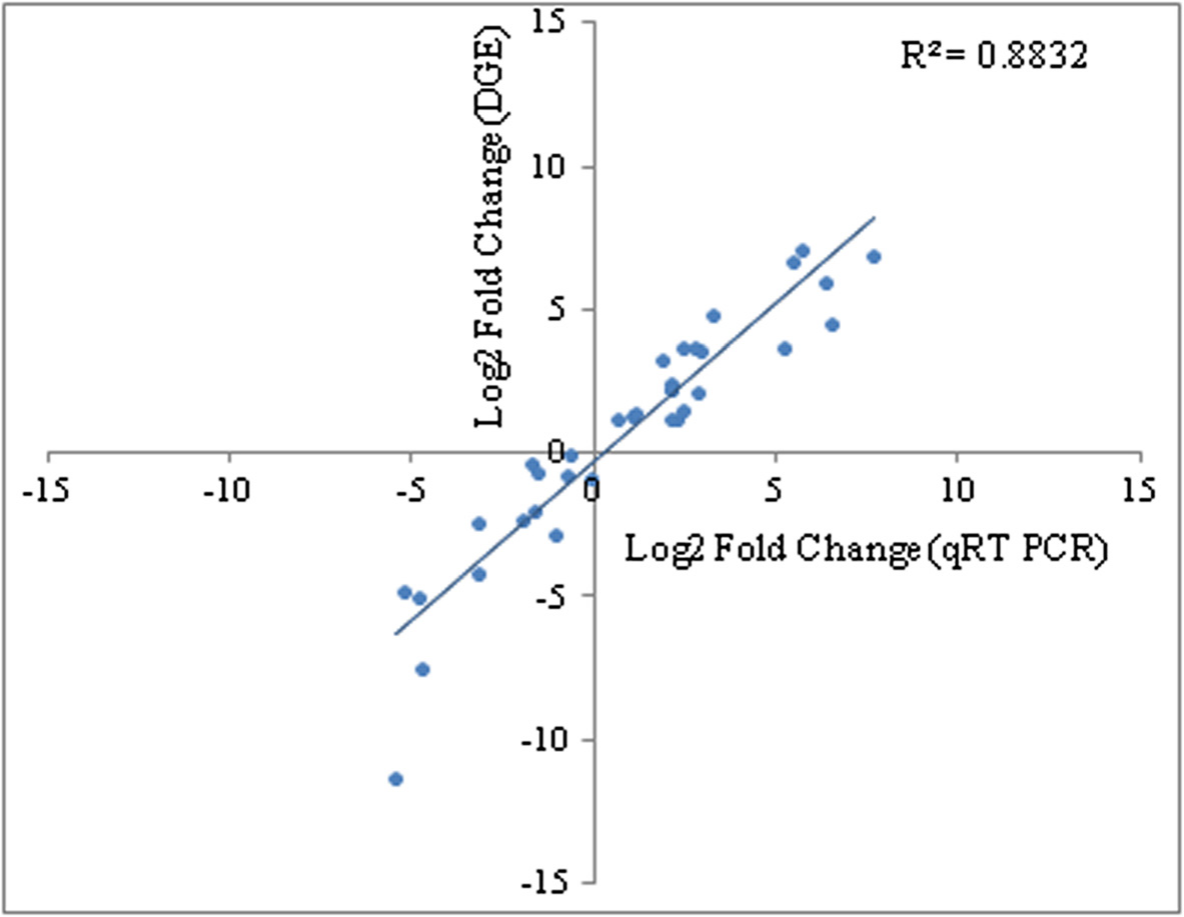
Correlation of DGE- and qRT-PCR-based expression profiles of transcripts. Expression profiles of 35 transcripts that are related to specialized metabolism were selected for the analysis. Data are presented as log2FoldChange (leaf vs root). qRT - PCR data represents average of three biological replicates. Differential transcript expression determined by qRT - PCR in root and leaf tissues was statistically evaluated either at *P*<0.05 or *P*<0.01

To generate a non-redundant reference transcriptome of kalmegh, leaf and root assembled transcripts were clustered using CD-HIT at 95 % sequence identity [34]. By following this approach, a total of 84,628 transcripts with an average length of 688.53 bp were generated (Table 2). For the identification of differential transcripts, these assembled unique transcripts (84,628) were considered as master control transcript and the read count profiles were first determined for the leaf and root Illumina reads using Bowtie tool [37] and further proceed for the differential gene expression analysis using DegSeq tool [38] (Additional file 1: Figure S2). This digital gene expression (DGE) analysis revealed 6277 and 5418 transcripts with expression only in leaf and root tissues, respectively. However, 19,564 and 16,678 transcripts were up-regulated and down-regulated, respectively, in leaf as compared to root. To authenticate DGE-based expression profiles of the transcripts, a correlation with quantitative RT-PCR (qRT-PCR)-based expression patterns was determined. For this, 35 transcripts that are related to the specialized metabolism were selected (Fig. 4). The overall correlation coefficient (r) of 0.9398 (r^2^ = 0.8832) indicated a very high level of correlation between DGE- and qRT-PCR-based expression profiles of the transcripts. Therefore, DGE-based expression profiles may be considered to identify transcripts involved in tissue-specific accumulation of specialized metabolites in kalmegh. To identify candidate transcripts of the *ent*-LRD biosynthetic pathway, transcripts were classified according to the DGE-based expression patterns in leaf and root tissues. DGE-based expression profiles of the transcripts with annotation to the diterpene biosynthetic pathway and to different CYP450, GT and transcription factor families are presented in Table 3; Additional file 1: Figure S4, Additional file 7: Table S6, Additional file 8: Table S7 and Additional file 9: Table S8.

**Table 2:** Combined assembly of leaf and root transcriptomes

Description	No.
Total number of contigs	8,4628
Average length (bp)	688.53
Total length (bp)	5,826,9163
Contigs (200–500 bp)	47,592
Contigs (501–1000 bp)	21,164
Contigs (1001–3000 bp)	14,528
Contigs (>3000 bp)	1344
N50 (bp)	976

**Table 3:** List of transcripts related to diterpene biosynthesis

Pathway	Transcript ID	Transcript annotation	Transcript length (bp)	Expression^a^
MEP	ApU56176	1-Deoxy-D-xylulose-5-phosphate synthase (DXS)	2421	1.210593385
	ApU12883	1-Deoxy-D-xylulose-5-phosphate synthase (DXS)	1284	−0.620028298
	ApU13057	1-Deoxy-D-xylulose-5-phosphate synthase (DXS)	2647	−0.064469923
	ApU57524	1-Deoxy-D-xylulose-5-phosphate synthase (DXS)	2529	4.485204599
	ApU8165	1-Deoxy-D-xylulose-5-phosphate reductoisomerase (DXR)	1019	2.090903695
	ApU50057	1-Deoxy-D-xylulose-5-phosphate reductoisomerase (DXR)	1084	2.274895363
	Ap2567	1-Deoxy-D-xylulose-5-phosphate reductoisomerase (DXR)	1013	4.853346
	ApU70472	2-C-Methyl-D-erythritol 4-phosphate cytidylyltransferase (MCT)	1277	3.71794589
	ApU7163	4-Diphosphocytidyl-2-C-methyl-D-erythritol kinase (CMK)	1553	1.228243799
	ApU3039	2-C-Methyl-D-erythritol 2,4-cyclodiphosphate synthase (MDS)	611	3.330961011
	ApU45802	(E)-4-Hydroxy-3-methylbut-2-enyl-diphosphate synthase (HDS)	2631	3.613822951
	ApU45495	(E)-4-Hydroxy-3-methylbut-2-enyl-diphosphate synthase (HDS)	2664	−0.839568655
	ApU67412	4-Hydroxy-3-methylbut-2-enyl diphosphate reductase (HDR)	1830	5.990336076
	ApU393	4-Hydroxy-3-methylbut-2-enyl diphosphate reductase (HDR)	1773	3.743023121
MEP/MEV	ApU9344	Isopentenyl-diphosphate delta-isomerase (IDI)	1165	−2.855299841
	ApU80862	Isopentenyl-diphosphate delta-isomerase (IDI)	426	Root only
MEV	ApU45787	Acetyl-CoA C-acetyltransferase (AACT)	758	0.073827478
	ApU3388	Acetyl-CoA C-acetyltransferase (AACT)	1132	−2.201783665
	ApU29957	Hydroxymethylglutaryl-CoA synthase (HMGS)	1009	−0.970530757
	ApU46957	Hydroxymethylglutaryl-CoA synthase (HMGS)	1127	−0.971997692
	ApU2925	Hydroxymethylglutaryl-CoA reductase (HMGR)	2235	1.932136157
	ApU46382	Hydroxymethylglutaryl-CoA reductase (HMGR)	2454	−2.018649251
	ApU51503	Mevalonate kinase (MK)	1202	−0.741169194
	ApU4232	Phosphomevalonate kinase (PMK)	1439	−1.11100184
	ApU58988	Phosphomevalonate kinase (PMK)	1171	−0.311783537
	ApU51812	Diphosphomevalonate decarboxylase (MVD)	573	−1.349116106
	ApU9903	Diphosphomevalonate decarboxylase (MVD)	974	−1.562474564
Diterpene	ApU8378	Geranylgeranyl diphosphate synthase (GGPS)	1749	1.018074228
	ApU952	Geranylgeranyl diphosphate synthase (GGPS)	984	2.233068085
	ApU55421	Geranylgeranyl diphosphate synthase (GGPS)	1522	1.364502354
	ApU53774	Ent-copalyl diphosphate synthase (Ent-CPS)	2623	Root only
	ApU55291	Ent-copalyl diphosphate synthase (Ent-CPS)	2567	−0.80001242
	ApU48901	Ent-copalyl diphosphate synthase (Ent-CPS)	2654	7.119944191
	ApU14593	Ent-kaurene synthase (KS)	1544	−1.728307905
	ApU66227	Ent-kaurene synthase (KS)	1707	−2.414805387
	ApU14229	Ent-kaurene oxidase (KO)	1966	−3.699209001
	ApU51425	Ent-kaurenoic acid hydroxylase (KAO)	1938	−6.870577872
	ApU51353	Ent-kaurenoic acid hydroxylase (KAO)	1911	−3.81097751
	ApU77665	Gibberellin 2-oxidase (GA2ox)	666	−4.234871361
	ApU79135	Gibberellin 2-oxidase (GA2ox)	628	−3.741831349
	ApU23389	Gibberellin 2-oxidase (GA2ox)	1330	4.108980573
	ApU10203	Gibberellin 20-oxidase (GA20ox)	635	3.315288446
	ApU51228	Gibberellin 20-oxidase (GA20ox)	1395	2.941320399
	ApU45906	Gibberellin 3-beta-dioxygenase (GA3ox)	1089	−5.116226864
	ApU57038	Momilactone-A synthase (MAS)	1003	−8.215762538
	ApU58121	Momilactone-A synthase (MAS)	846	−9.795908022
	ApU67465	Momilactone-A synthase (MAS)	972	Root only
	ApU1116	Momilactone-A synthase (MAS)	1145	2.550321912

^a^log2FoldChange (leaf vs root) based on DGE

### Transcripts of the diterpene biosynthetic pathway

**Fig. 5:**
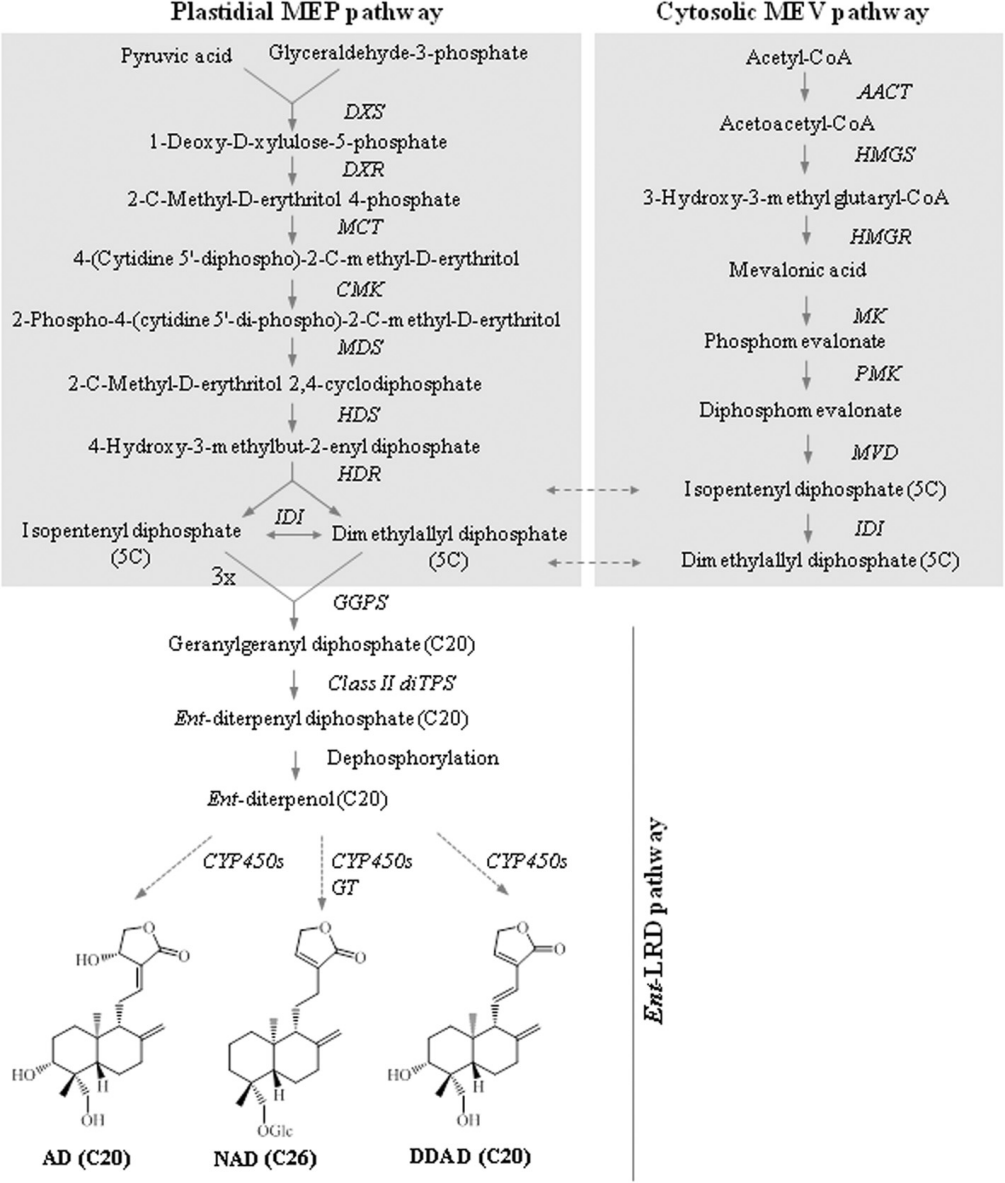
Proposed pathway for *ent*-LRD biosynthesis in kalmegh. Putative transcripts of the pathway and corresponding enzymatic steps are shown. AD, andrographolide; NAD, neoandrographolide; DDAD, 14-deoxy-11,12-didehydroandrographolide

In plants, plastidial 2C-methyl-D-erythritol-4-phosphate (MEP) and cytosolic mevalonic acid (MEV) pathways provide two 5C isoprenoid building blocks, dimethylallyl diphosphate (DMAPP) and isopentenyl diphosphate (IPP), for the biosynthesis of diverse terpene metabolites [43]. IPP and DMAPP derived from the MEP pathway are converted to monoterpenes, diterpenes, and tetraterpenes, whereas those derived from the MEV pathway are converted to sesquiterpenes and triterpenes. However, cross-talk between these two pathways in biosynthesis of some terpenes was also recognised [44–46]. Previously, a major role of the MEP pathway and a minor role of the MEV pathway for supplying the 5C isoprenoid precursors for the biosynthesis of andrographolide were reported [47]. Transcripts predicted to encode all the enzymes of the MEP and MEV pathways are identified in kalmegh transcriptome (Table 3; Fig. 5); further demonstrating the quality and in-depth coverage of the transcriptome database generated in this study. Interestingly, four transcripts for DXS, three for DXR and two each for HDS, HDR, AACT, HMGS, HMGR, PMK, MVD and IDI were revealed in kalmegh transcriptome. This observation suggests the likely existence of multiple isomers for these enzymes in kalmegh.

**Fig. 6:**
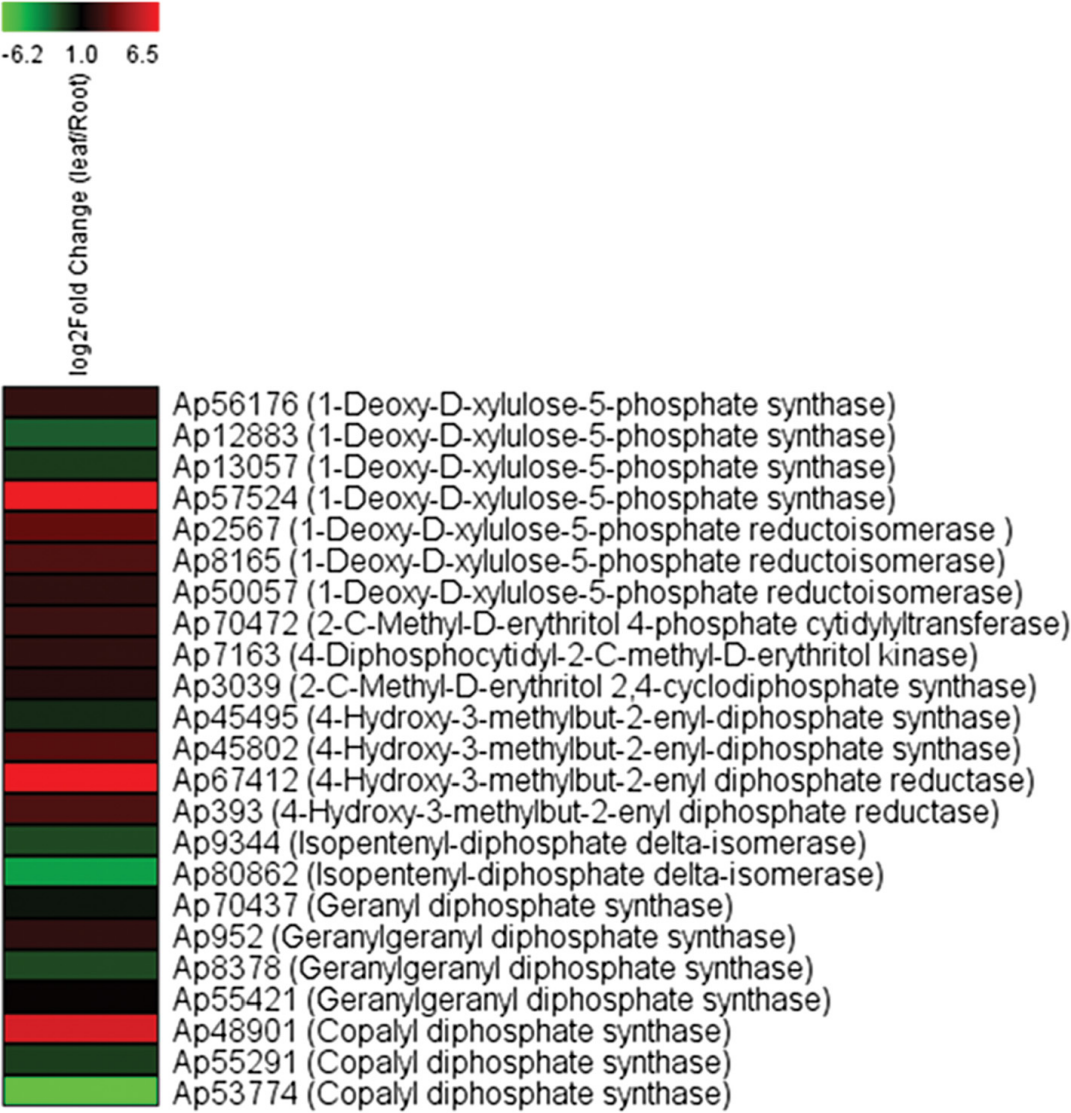
Relative expression level of transcripts, putatively related to *ent*-LRD biosynthesis. Expression profiles of the MEP pathway transcripts, GGPSs and class II diTPSs were determined through qRT - PCR in root and leaf tissues. Data are presented as log2FoldChange (leaf vs root) obtained from three biological replicates. Differential transcript expression in root and leaf tissues was statistically evaluated either at *P*<0.05 or *P*<0.01

The second stage of diterpene biosynthesis involves head-to-tail condensation of three IPP and one DMAPP to a C20 compound geranylgeranyl diphosphate (GGPP) (Fig. 5). This prenyltransfer reaction is catalyzed by the plastidial geranylgeranyl diphosphate synthase (GGPS). In *ent*-LRD biosynthetic pathway, GGPP is further cyclized into *ent*-diterpenyl diphosphate e.g., *ent*-copalyl diphosphate (*ent*-CPP) following protonation-initiated cyclization mechanism catalyzed by the class II diterpene synthase (diTPS). *Ent*-diterpenyl diphosphate then acts as substrate for the class I diTPS that catalyzes further cyclization and/or rearrangement reactions [48, 49]. Thus, based on structures of kalmegh *ent*-LRDs, the involvement of class I and class II diTPSs, CYP450s and GTs enzymes in the biosynthesis of *ent*-LRDs was hypothesized (Fig. 5). From kalmegh transcriptome database, three partial transcripts for the GGPS and three full-length transcripts for class II diTPSs with homology to the *ent*-copalyl diphosphate synthase (*ent*-CPS) are identified (Table 3). Besides, several transcripts for CYP450s and GTs are also recognized (Fig. 5; Additional file 7: Table S6 and Additional file 8: Table S7). Transcripts that encode MEP pathway enzymes, GGPS, class II diTPS, CYP450 and GT, and preferentially expressed in leaf tissue (Fig. 6; Table 3; Additional file 7: Table S6, Additional file 8: Table S7 and Additional file 1: Figure S4) are potential candidates for the biosynthesis of *ent*-LRDs in kalmegh. Although, two class I diTPSs with sequence similarity with *ent*-kaurene synthase are identified (Table 3), none of them preferentially expressed in leaf. Thus, their involvement in the biosynthesis of *ent*-LRD medicinal compounds in kalmegh may be excluded.

In contrast to diTPSs, not many CYP450s and GTs of specialized diterpene biosynthetic pathways are known. Some of the characterized members include CYP450s of taxol, phytoalexins and diterpene resin acid, and GTs of steviol glycoside biosynthetic pathways [50–56]. The majority of CYP450s of the specialized terpene metabolism belong to the CYP71 and CYP85 clans [57, 58]. From kalmegh transcriptome database, 147 transcripts that belong to the clan 71 (CYP families 71, 76, 78, 81–84, 93, 98, 706, 736) and clan 85 (CYP families 85, 90, 707, 716) are identified (Additional file 7: Table S6 and Additional file 1: Figure S4). Among these, 45 transcripts preferentially expressed in leaf compared to root. On the other hand, family 1 GTs (GT1) are the key players in glycosylation of specialized metabolites [59, 60]. Among the total of 161 GT1s of kalmegh, 55 GT1s preferentially expressed in leaf compared to root (Additional file 8: Table S7 and Additional file 1: Figure S4). Further studies on these leaf-expressed CYP450s of the clan 71 and clan 85, and GT1s can lead to the identification of potential oxidase(s) and GT(s) of kalmegh *ent*-LRD biosynthetic pathway.

### Identification of simple sequence repeats in diterpene biosynthetic pathway transcripts

Simple sequence repeats (SSRs) are often considered most efficient and reliable molecular markers for detecting genetic variations in plants [61]. Therefore, to identify functional SSRs of kalmegh, leaf and root transcripts were examined for the presence of microsatellite motifs using MIcroSAtellite (MISA) tool (http://pgrc.ipk-gatersleben.de/misa). A total of 16,485 potential SSRs were identified in 13,805 leaf transcripts (Additional file 10: Table S9). Whereas, 15,911 SSRs were detected in 13,213 root transcripts. Moreover, 2194 leaf and 2200 root transcripts were detected with more than one SSRs. Di-nucleotide repeats were the most abundant SSRs in leaf and root transcripts with 5194 and 5023 SSRs, respectively. The numbers of compound SSRs were 1877 and 1895 in leaf and root transcripts, respectively. The complete lists of SSRs detected in leaf and root transcripts are provided in Additional file 11: Table S10 and Additional file 12: Table S11. Interestingly, several SSRs were also identified in transcripts of the specialized metabolic pathways, including terpenes and phenylpropanoids (Additional file 13: Table S12). SSRs were detected for the transcripts of the MEP pathway enzymes (DXS, MDS and HDR), GGPS and class II diTPSs (ApCPS2, ApCPS3). These SSRs could be useful in genotyping cultivars and developing specific chemotypes of kalmegh following marker-assisted selection.

### Identification and analysis of diterpene synthases

**Fig. 7:**
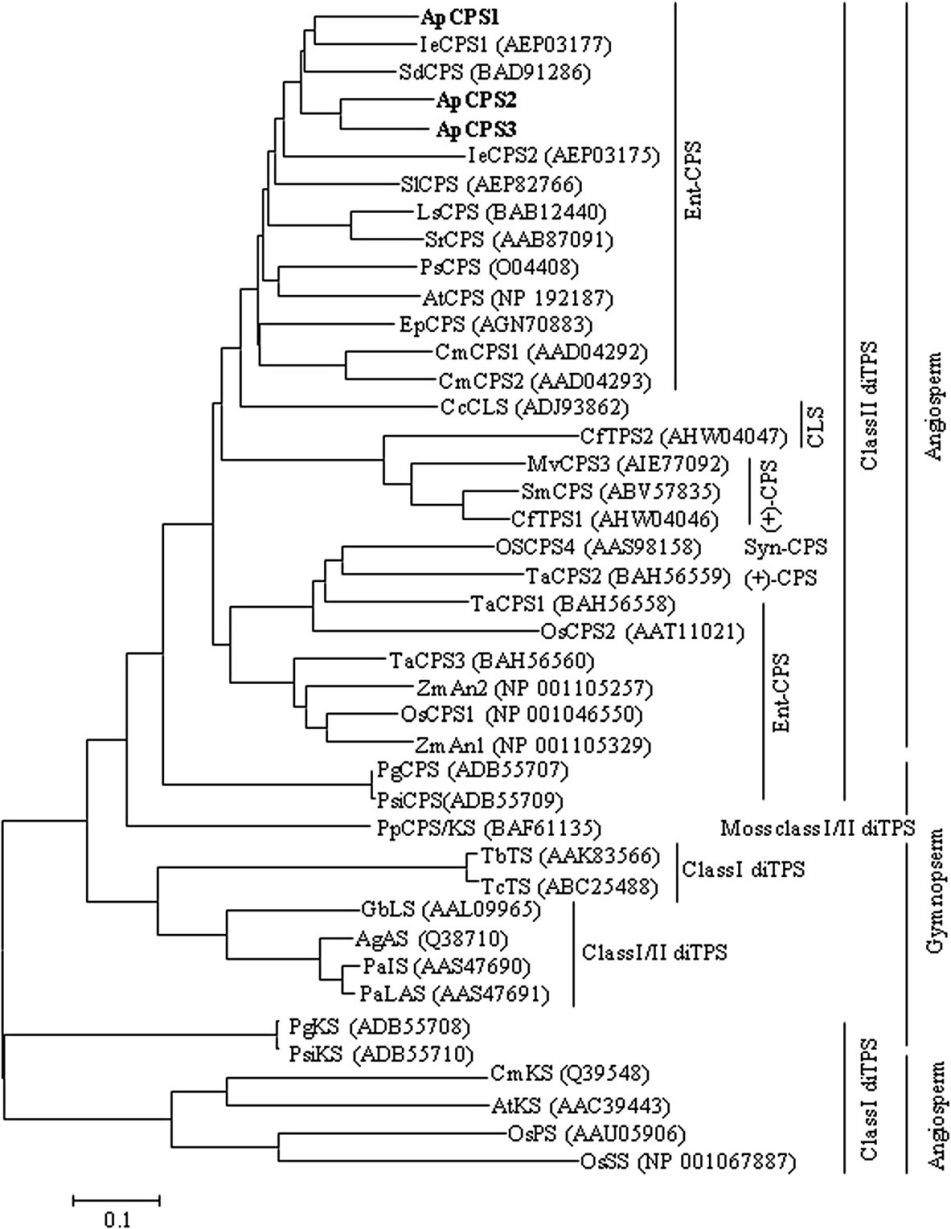
The phylogenetic relationship of kalmegh class II diTPSs with diTPSs of angiosperms, gymnosperms and moss. The evolutionary history was inferred using the Neighbor-Joining method. The evolutionary distances were computed using the Poisson correction method and are in the units of the number of amino acid substitutions per site. Evolutionary analyses were conducted in MEGA6. *Ent-*CPS, *syn-*CPS*,* (+)-CPS denote *ent-, syn-* and (+)/normal copalyl diphosphate synthase, respectively. CLS means copal-8-ol diphosphate synthase

Annotation of the kalmegh transcriptome revealed three diTPSs that showed close phylogenetic relationship with the dicotyledons monofunctional class II diTPSs of *ent*-CPP product specificity (Fig. 7). These are ApCPS1 (ApU55291), ApCPS2 (ApU48901) and ApCPS3 (ApU53774) (Table 3). Similar to class II diTPSs, the highly conserved DXDD motif that is essential for the protonation-initiated cyclization of GGPP was identified in ApCPS1, ApCPS2 and ApCPS3, following multiple sequence alignment (Additional file 1: Figure S5). Sequence analysis revealed that *ApCPS1*, *ApCPS2* and *ApCPS3* encode for 832-, 817- and 797- amino acids proteins with calculated molecular masses of 95.45, 93.43 and 90.81 kD, respectively. At the amino acid sequence level, ApCPS1 shared 55.2 and 57.21 % identities with ApCPS2 and ApCPS3, respectively (ClustalW score, http://www.ebi.ac.uk/). However, ApCPS2 shared 63.36 % amino acid identity with ApCPS3. Like other plant diTPSs, N-terminal transit peptides for the chloroplast localization were recognised in *ApCPS1*, *ApCPS2* and *ApCPS3*, following iPSORT (http://ipsort.hgc.jp/) and Predotar v. 1.30. (http://urgi.versailles.inra.fr/predotar/predotar.html) analysis.

**Fig. 8:**
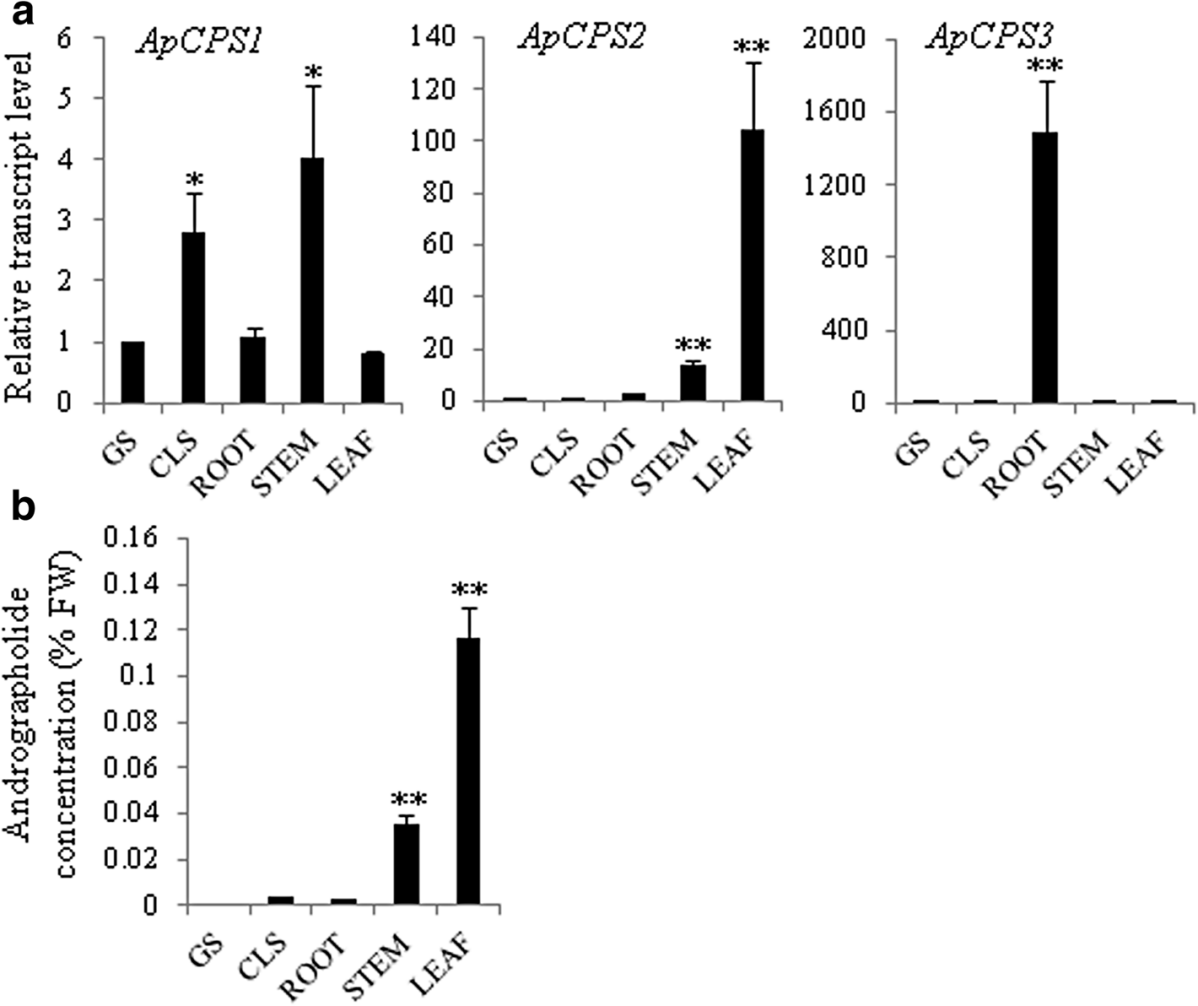
Tissue-specific expression profiles of class II diTPSs and accumulation pattern of major *ent*-LRD andrographolide. **a** Transcript levels were determined by qRT-PCR analysis. Data are presented as the mean (±SE) of three biological replicates. **b** Andrographolide levels in different tissues were determined by high-performance liquid chromatography analysis. Data are presented as the mean (±SE) of three biological replicates. Andrographolide was either undetected or detected at very low level in GS, CLS and root samples. *P<0.05 and **P<0.01 compared with GS sample

*ApCPS1*, *ApCPS2* and *ApCPS3* exhibited dissimilar expression patterns in leaf and root tissues (Fig. 6). The transcripts levels of *ApCPS1* were comparable in leaf and root tissues. However, *ApCPS2* showed high level of transcript accumulation in leaf and low level of transcript accumulation in root. In contrast, *ApCPS3* transcripts were detected at very high level in root and at very low level in leaf. This divergent expression pattern of *ApCPS1*, *ApCPS2* and *ApCPS3* indicated their role in different diterpene metabolic pathways of kalmegh, although, their involvement in same biosynthetic pathway with functional redundancy cannot be completely excluded. In order to determine potential functions of *ApCPS1*, *ApCPS2* and *ApCPS3* in kalmegh, transcripts levels were analysed in different plant organs and during seedling development stages following qRT - PCR (Fig. 8a). Moreover, to correlate transcript expression with metabolite accumulation pattern, the level of andrographolide, the most abundant *ent*-LRD of kalmegh, was determined in plant organs and during seedling developmental ages following HPLC analysis (Fig. 8b and Additional file 1: Figure S6). Maximum transcript level for *ApCPS1* was detected in stem (4.03-fold), followed by seedlings at cotyledonary leaf stage (CLS, 2.79-fold) as compared to germinating seeds (GS). *ApCPS1* transcript was also detected during seed germination. Because *ent*-CPP also serves as precursor for the biosynthesis of phytohormone gibberellin (GA) that is known to promote seed germination, seedling development and stem elongation in plant species [48, 49, 62, 63], we suggest the role of *ApCPS1* in general metabolism by providing *ent*-LRD precursor for GA biosynthesis. In contrast to *ApCPS1*, *ApCPS2* transcript expression was maximum in leaf (104.34-fold), followed by stem (14.19-fold) as compared to GS. However, very low level of *ApCPS2* transcript was detected during seed germination and in seedlings at the CLS stage, as compared to leaf and stem. Based on the transcript expression and *ent*-LRD metabolite accumulation patterns in plant organs and during seedling developmental ages, the role of *ApCPS2* in tissue-specific accumulation of medicinal *ent*-LRDs was anticipated (Fig. 8a and b). Although, bioactive *ent*-LRDs accumulate at high level in leaf (86.84-fold compared to GS), they were undetected or detected at very low level in root [41] (Fig. 8b). The high level expression of *ApCPS3* in kalmegh root (1491.41-fold as compared to GS) suggests biosynthesis of LRD(s) in root which is/are yet to be identified. The role of *ApCPS3* in root diterpene phytoalexin biosynthesis cannot be excluded. We hypothesized this function of *ApCPS3* because class II diTPSs are known to play role in root phytoalexin biosynthesis in plants [64–66]. Moreover, kalmegh transcripts putatively encoding momilactone-A synthase, a phytoalexin biosynthetic pathway enzyme [67], also expressed at high level in roots (Table 3).

In medicinal *ent*-LRDs biosynthetic pathway of kalmegh, *ent*-diterpenyl diphosphate/*ent*-CPP produced from class II diTPS activity might acts as substrate of class I diTPS for further hydrolysis of the phosphate group without additional cyclization and rearrangement steps. This class I diTPS activity may be related to the class I diTPS activity of the bifunctional class I/II diTPS of *Selaginella moellendorffii* [68]. However, transcripts that show sequence similarity to the class I diTPSs and preferentially express in leaf tissue which accumulates medicinal *ent*-LRDs, could not be identified. Although two transcripts for class I diTPSs with sequence homology to the *ent*-kaurene synthase are recognised (Table 3), both of them preferentially expressed in root compared to leaf and have a stretch of overlapping sequences; suggesting that the two contigs might in fact represent a single transcript. Therefore, the role of an endogenous phosphatase for the conversion of *ent*-diterpenyl diphosphate*/ent*-CPP into *ent*-diterpenol/*ent*-copalol is also likely, as was shown for the biosynthesis of diterpenol in *Nicotiana* [69, 70]. Additional structural diversities in *ent*-LRDs including oxygen functionality and glycosylation might be brought about by the activities of the CYP450s and GTs, respectively, that preferentially express in leaf tissue (Additional file 7: Table S6, Additional file 8: Table S7 and Additional file 1: Figure S4).

## Conclusion

Several bioactive specialized metabolites, including *ent*-LRDs were isolated from kalmegh [11, 12]; however, their biosynthesis was not studied. The present study was undertaken with the aim to identify candidate genes involved in the biosynthesis of specialized metabolites with special emphasis on *ent*-LRDs that are considered as the major medicinally active components of kalmegh. Independent sequencing of leaf and root transcriptomes using Illumina HiSeq2000 platform and individual as well as combined assembly of the transcriptomes resulted in generation of a reference transcriptome of kalmegh with in-depth coverage. This experimental approach also helped us to gather information regarding expression patterns of the identified transcripts in *ent*-LRD accumulating and non-accumulating tissues. Transcripts predicted to encode all the enzymes of the MEP and MEV pathways, GGPSs, diTPSs, CYP450s and GTs are identified and classified, and based on transcript expression patterns, their role in the tissue-specific accumulation of medicinal *ent*-LRDs is discussed. Our results indicate the occurrence of three isoforms for the class II diTPS (*ApCPS1*, *ApCPS2* and *ApCPS3*) in kalmegh. These genes showed discrete spatio-temporal expression patterns suggesting their participation into distinct diterpene metabolic pathways of kalmegh. Data suggest the role of *ApCPS1* in general metabolism (GA biosynthesis); while *ApCPS2* is potentially involved in the biosynthesis of medicinal *ent*-LRDs in leaf. In contrast, expression pattern of *ApCPS3* suggests its involvement in the biosynthesis of root diterpenes, possibly phytoalexins. In addition, SSRs were identified in the transcripts of the specialized metabolic pathways, including *ent*-LRDs. These SSRs might be useful in selecting and developing desired chemotypes of kalmegh following molecular breeding approaches. Taken together, these results will help us to understand the molecular and regulatory basis of tissue-specific accumulation patterns of medicinally active specialized metabolites in kalmegh and to develop molecular breeding strategies to improve their yields.

## Additional files

Additional file 1: Figure S1. Flow chart displaying strategies employed for *de no*vo transcriptome analysis. **Figure S2.** Flow chart displaying strategies employed for digital gene expression analysis. **Figure S3.** Gene ontology classification of root and leaf transcripts. **Figure S4.** Transcription factor, cytochrome P450 monoxygenase and glycosyltransferase families. **Figure S5.** Amino acid sequence comparison of class II diTPSs of kalmegh. **Figure S6.** HPLC chromatograms of metabolites extracted from anderographolide-accumulating (Leaf and stem) and non-accumulating (root, GS, CLS) tissues of *A. paniculata*. (PDF 367 kb) Additional file 2: Table S1. List of the primers used in this study. (DOCX 20 kb) Additional file 3: Table S2. Annotation of leaf transcripts to the Uniprot database. (XLS 17060 kb) Additional file 4: Table S3. Annotation of root transcripts to the Uniprot database. (XLS 16520 kb) Additional file 5: Table S4. KEGG pathways represented by leaf transcripts. (XLS 719 kb) Additional file 6: Table S5. KEGG pathways represented by root transcripts. (XLS 710 kb) Additional file 7: Table S6. Putative cytochrome P450 monooxygenases. (XLSX 47 kb) Additional file 8: Table S7. Putative Glycosyltransferases. (XLS 195 kb) Additional file 9: Table S8. Putative transcription factors. (XLSX 45 kb) Additional file 10: Table S9. A summary of SSRs identified in leaf and root transcriptomes. (DOCX 11 kb) Additional file 11: Table S10. SSRs in leaf transcripts. (XLS 2660 kb) Additional file 12: Table S11. SSRs in root transcripts. (XLS 1818 kb) Additional file 13: Table S12. SSRs in specialized metabolic pathway transcripts. (XLSX 13 kb)
