# Supplementary material for: Andrographis paniculata transcriptome provides molecular insights into tissue-specific accumulation of medicinal diterpenes
Source: BMC Genomics. 2015 Sep 2;16(1):659. doi: 10.1186/s12864-015-1864-y (PMC4557604; doi:10.1186/s12864-015-1864-y)
Supplement: Additional file 1: Figure S1. — Flow chart displaying strategies employed for de novo transcriptome analysis. Figure S2. Flow chart displaying strategies employed for digital gene expression analysis. Figure S3. Gene ontology classification of root and leaf transcripts. Figure S4. Transcription factor, cytochrome P450 monoxygenase and glycosyltransferase families. Figure S5. Amino acid sequence comparison of class II diTPSs of kalmegh. Figure S6. HPLC chromatograms of metabolites extracted from anderographolide-accumulating (Leaf and stem) and non-accumulating (root, GS, CLS) tissues of A. paniculata. (PDF 367 kb) [file 12864_2015_1864_MOESM1_ESM.pdf]

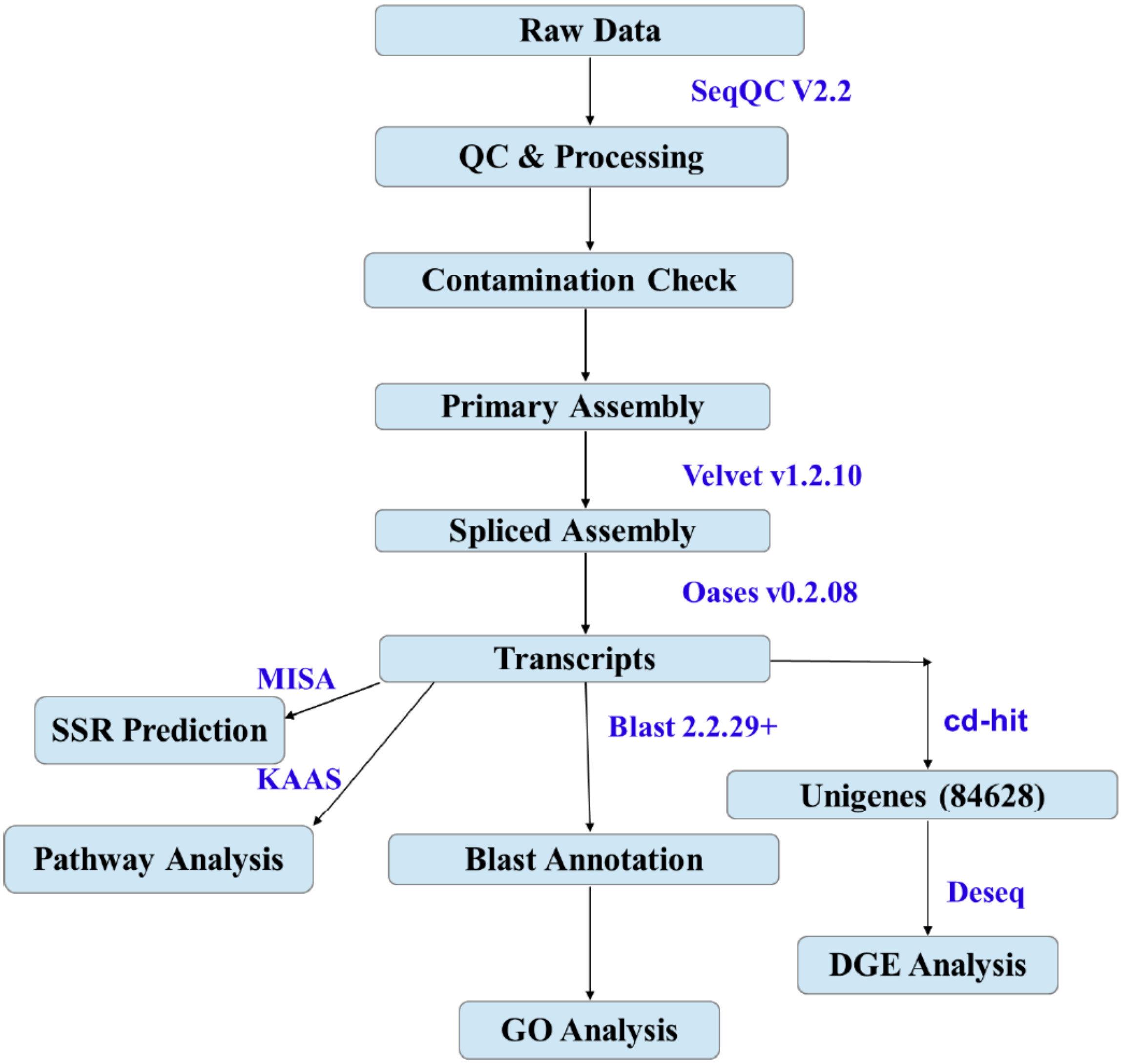

Supplementary Figure S1. Flow chart displaying strategies employed for *de no*vo transcriptome analysis.

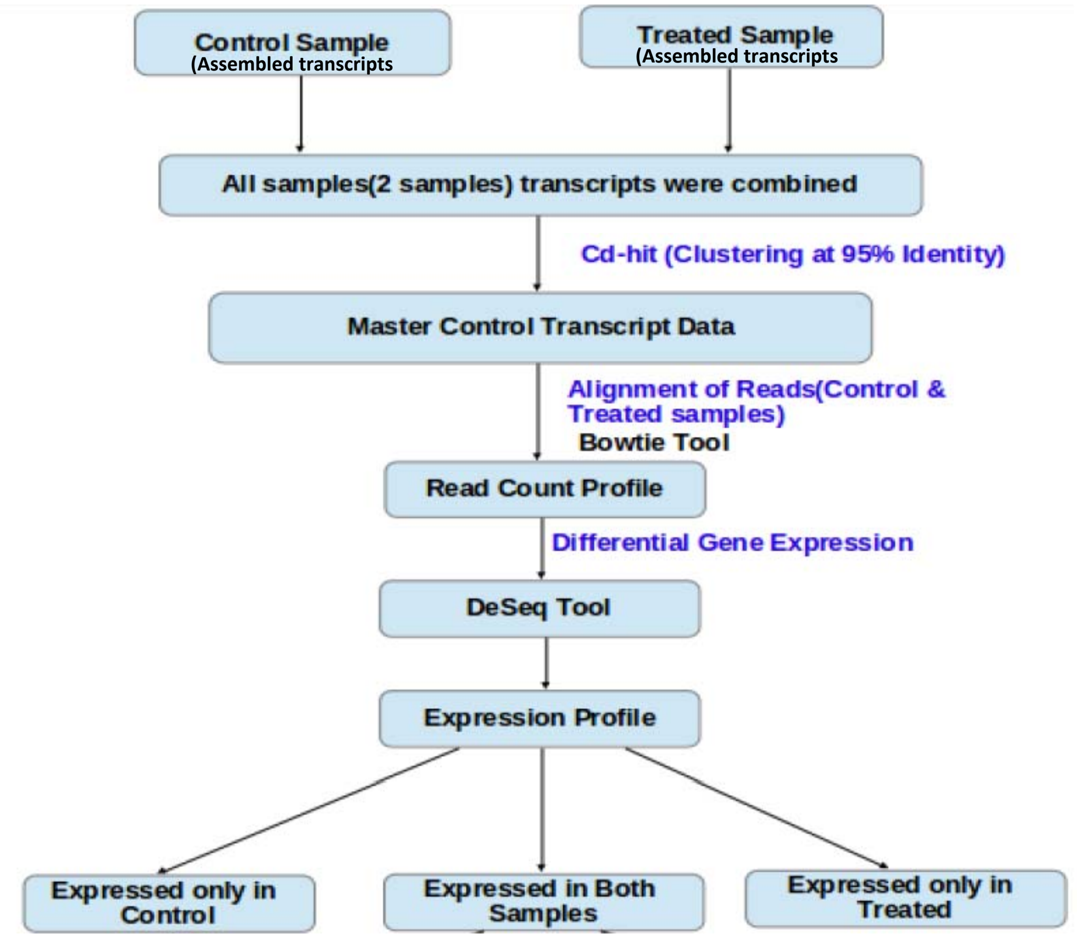

Supplementary Figure S2. Flow chart displaying strategies employed for digital gene expression analysis.

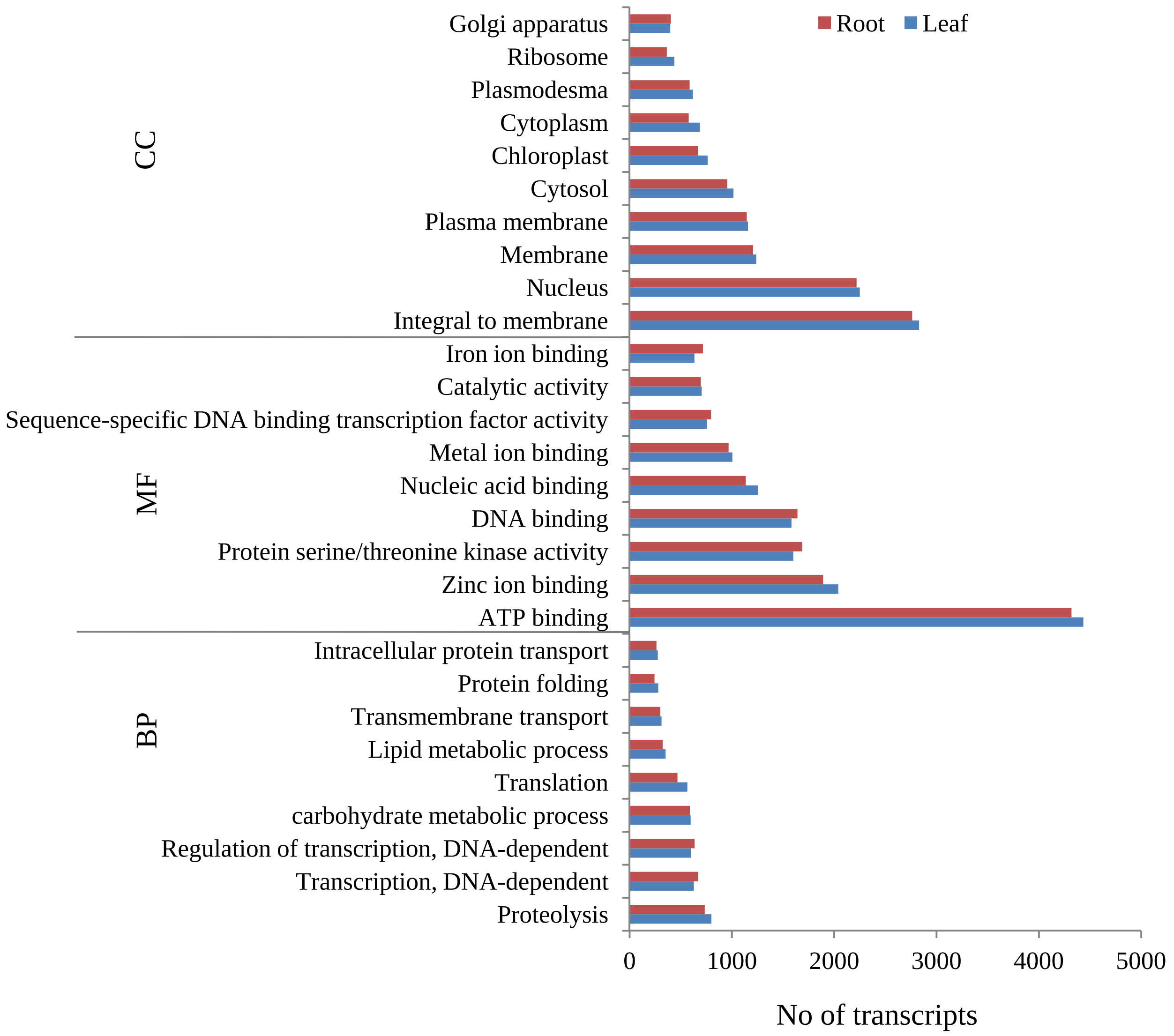

Supplementary Figure S3. Gene ontology classification of root and leaf transcripts. BP- biological process, MF- molecular function, CC- cellular component.

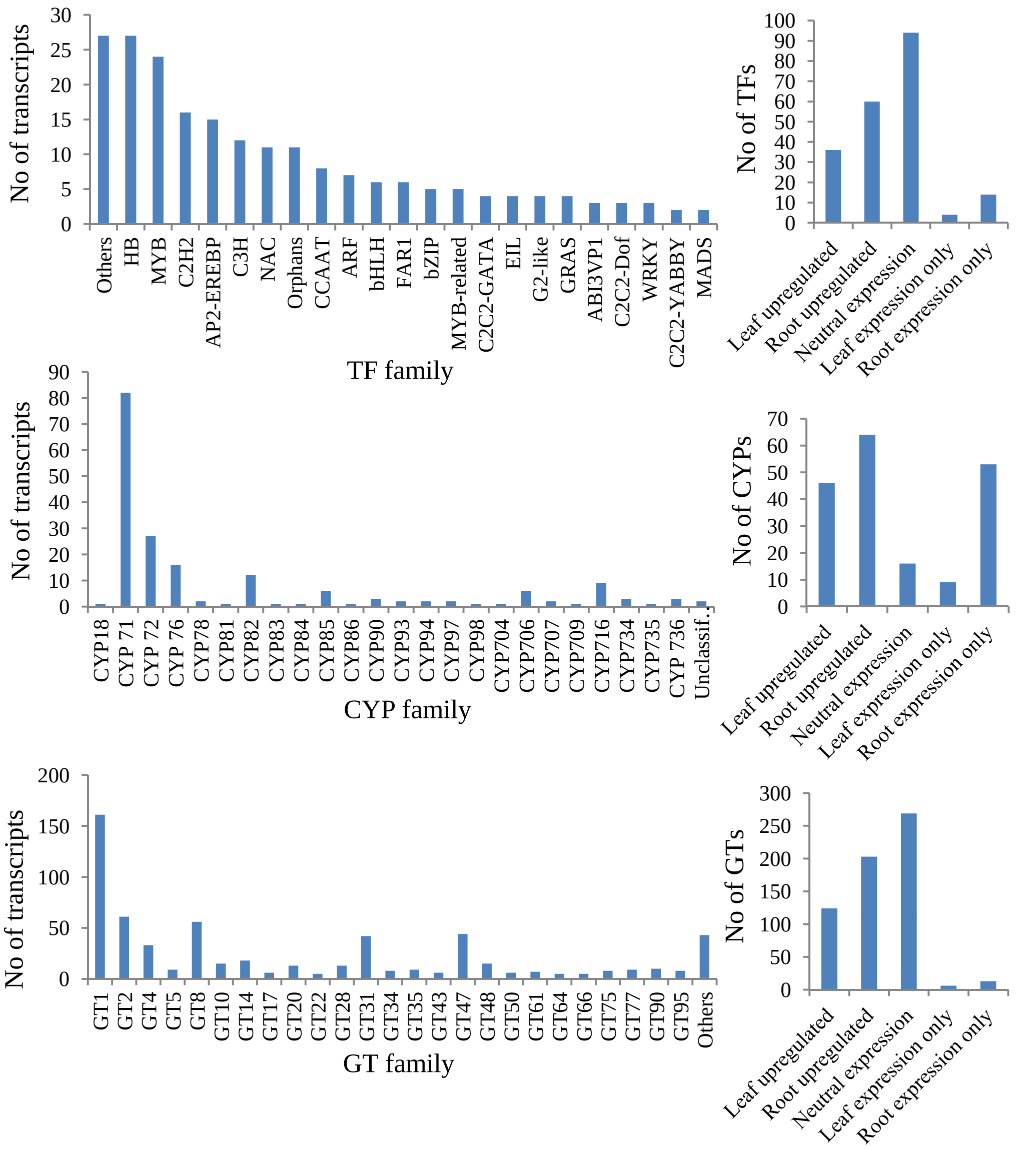

Supplementary Figure S4. Transcription factor, cytochrome P450 monoxygenase and glycosyltransferase families. Expression patterns of the transcripts are based on DGE. Leaf upregulated, root upregulated and neutral expression denote log2FoldChange (leaf vs root) of ≥1, ≤-1 and between 1 and -1, respectively.

```
ApCPS2          --MSSLLFHQPLCLHQPLHRRGSPHALS--TAGAFQYLKSVGST-CRILECNAAYKPHAE 55
ApCPS3          --MASAFAPLQSLLPSTLLGASPASRRA--AAGVFQPSKASG---LSTFQCRAVQKSQAE 53
ApCPS1          MPLFSLLGSSPSPPPQLYIPAASPFPRTSVVAGLFSVWRNTTNTGYPLLQCGAVSRAPAE 60
                  : * :        .     ...   :  .** *.  :         ::* *. :. **

ApCPS2          -ELVGAIQNELPDTKLWEQIVEDGTEEDD----SIFLDSDKIRKQVQLIQSMLQSMGDGE 110
ApCPS3          -SLDLPVKYYWQQKTAVEENDRHEEEDAL----QESKESDKVRNHVELIRSMLQSMGDGE 108
ApCPS1          GEYIDVILSGFPVRKWPSNIATEGDTQKDKYKGEEVRSNERRDRMVEMIKSMLRSMDDGE 120
                 .    :       .  .:   .   :      .    ..::  . *::*:***:**.***

ApCPS2          ITVSPYDTAWVAMVEDINSADRVPQFPSSVDWISNNQLSDGSWGDEYIFSIYDRIINTLG 170
ApCPS3          ITVSPYDTAWVAMVEDIAGGG-KPQFPSSLDWISGNQLSDGSWGDAEIFSIYDRIINTLG 167
ApCPS1          ISISPYDTAWVALVEDIGGKG-QPQFPTSIEWISNNQLEDGSWGDSAAYSAHDRILNTLA 179
                *::*********:****  . .  ****:*::***.***.******    :* :***:***.

ApCPS2          CVAALRSWNIHPEKSEKGISFIKANIHKLEDENEEHMPIGFEVALPSLLEIAKKLDIDIP 230
ApCPS3          CVAALKTWNLHPHQAQKGISFIKENIHKLQDENEEHMPIGFEVAMPSLMDIAKKLDIDIP 227
ApCPS1          CVVALRSWKMHQDKTDKGVTFIRKNIHRLDEEKEEHMPIGFEVALPSLIETAKKLGIDVP 239
                **.**::*::* .:::**::**: ***:*:*::************:***:: ****.**:*

ApCPS2          YDAPCLQEIYRRRDIKLKKIPKNILHKVPTTLLHSLEGMAD-LEWDKLIKLQCADGSLLF 289
ApCPS3          YEAPALQEIYQRREIKLKKIPRQVMHTVPTTLLHSLEGMPD-LVWEKLLKLQSSNGSFLF 286
ApCPS1          NDSPALQKIYARRELKLTRIPRDIMHKVPTTLLHSLEGMSSGLEWQELLKLQCPDGSFLF 299
                 ::*.**:** **:**:**.:**::::*.************.. * *::*:***..:**:**

ApCPS2          SPSSTAFALQQTKDQNCLKYLDKAVQKFNGGVPNVYPVDLFEHIWVVDRLQRLGISRYFK 349
ApCPS3          SPSSTAFALQQTKDEKCLGYLGNMVQKFNGGVPNVYPVDLFEHIWAVDRLQRLGISRYFQ 346
ApCPS1          SPSSTAFALQQTKDENCLKYLLKHVRKFNGGVPNVYPVDLFERLWAVDRLQRLGISRYFQ 359
                **************::** ** : *:****************::*.**************:

ApCPS2          SELQESIDYVHRHWTNKGICWARNSEVQDIDDTAMAFRLMRLYGYKISADVFKNFQNGDE 409
ApCPS3          SEIDECVHYVHRYWTNRGICWARDSEVQDIDDTAMAFRLMRMQGYEVSSDVFKNFMRGGE 406
ApCPS1          PEIEECLGYVHRYWTDKGICWARNSQVQDVDDTAMGFRLLRLHGYRVSADVFKNFKQDEE 419
                .*::*.: ****:**::******:*:***:*****.***:*:*:*.:*:****** .. *

ApCPS2          FFCFSGQSTQAVTGMYNLYRASQLRFPGEDILIQAKNFSAKYLQEKRANNELLDKWIITK 469
ApCPS3          FFCFVGQSSQAVTGMYNLYRASQLMFPGEDILSDARKFSAKYLQEKRADNQLLDKWIITK 466
ApCPS1          FFCFAGQSNQAVTGMYNLYRASQVMFPGEVILAEARKFSHKFLQEKRANNELLDKWIITK 479
                **** ***.**************: **** ** :*::** *:******:*:*********

ApCPS2          DLPGEVSYALDVPWYANLPRVETRFYLEQYGGYEDVWIGKTLYRMPYVNNNTYLELAKLD 529
ApCPS3          DLPGEVGYALDVPWYASLPRLETRFFLDQYGGHDDVWIGKTLYRMPYVNNDTYLELAKLD 526
ApCPS1          DLPGEVGFALDIPWYASLPRIETRFFLEQYGGGDDVWIGKTLYRMPYIDNRAYLDLAKLD 539
                ******.:***:****.***:****:*:**** :*************::* :**:*****

ApCPS2          FNNCQALHREELRNLQKWSRNCSLFEFGLTDATLLQTYHIVSASVFEPERSAERIAWAKT 589
ApCPS3          YSNCQALHQDELRSIQKWCRKFSLAEFGLTGESLLQAYYIAAASIFAPEQALERLTWAKT 586
ApCPS1          YNNCQALHQSEWKSFQKWYRSCRLEEFGLSETTLVQTYYIAAASIFEPERLYERLAWAKT 599
                :.******:.* :.:*** *.  * ****:  :*:*:*:*.:**:* **:  **::****

ApCPS2          AILVETIVSHFHQNHFSPHQISTFVHEFANTTSPNSGSQNNGSPR-RISTFVGAIVRTLN 648
ApCPS3          VILVETLVSHFDHG--SKEQKLDFVNEFELAWS--SPKYNNGARYNTKSIFGAMMIRAFN 642
ApCPS1          AILMETVMRHSDQKKLSKQQKHALVNEFKHCAL-------LGERYKTRNNLIGTLVRSVN 652
                .**:**:: * .:   * .*   :*:**              *       . : . ::*:.*

ApCPS2          QLSLDMLSAHGRHVHPQIVHAWQSWLDTWTNQEGDSSSRHGEAELYLRVLNISS---ESG 705
ApCPS3          QISLDIQLAHCPDDHNRFNDAWRNWLNKWIEAEDDP--RQGEAELQIEILNLSS---CAT 697
ApCPS1          ELSLDAPLARYADIQSHLHRAWQKWLSSWEEGDMGE----GDAELLVCTLNLCGGGRNSH 708
                ::***   *:  .  : ::  **:.**..* : : .     *:*** :  **:..    :

ApCPS2          SLEDHDLLKSHPKYQRLLAITESVCSKLRLFQTQKDRGEAMCG--QDGTTTAEIESEMEE 763
ApCPS3          SQELSPLLHS--EYQQLMKATVAVCDQLHLHQSLKDAN--------GGISTAEIESSMQQ 747
ApCPS1          SRWSDKLLLSYPPYQPLVQIASRVCHHLRLSPTRKEEARMCEQKLQSGVSGGDIEGSMQE 768
                *     ** *   ** *:  :  ** :*:*  :  *:          .* : .:**..*::

ApCPS2          LVEMVVVKDDDDDDLESGVKQKFLTVSRSFYYSAYCNPGTISFHIAKVLFEKVL------ 817
ApCPS3          LAKLVLKKSS----MDSALKKNFLTVARSFYYRSYFGPETINSHIDKVLFERVL------ 797
ApCPS1          LVRLVLAKSHS--ELEFRVKQNFLLVARSYYYTAYCNPATINLHIAKVLFETVVLECCDD 826
                *..:*: *.      ::  :*::** *:**:** :* .* **. ** ***** *:

ApCPS2          ------
ApCPS3          ------
ApCPS1          HTRRYF 832
```

Supplementary Figure S5. Amino acid sequence comparison of class II diTPSs of kalmegh. Multiple sequence alignment was carried out by using ClustalW2 program (http://www.ebi.ac.uk/Tools/msa/clustalw2/). Identical amino acid residues are depicted as *, while conserved and semi-conserved substitutions are marked as : and ., respectively. Conserved DXDD motif is highlighted.

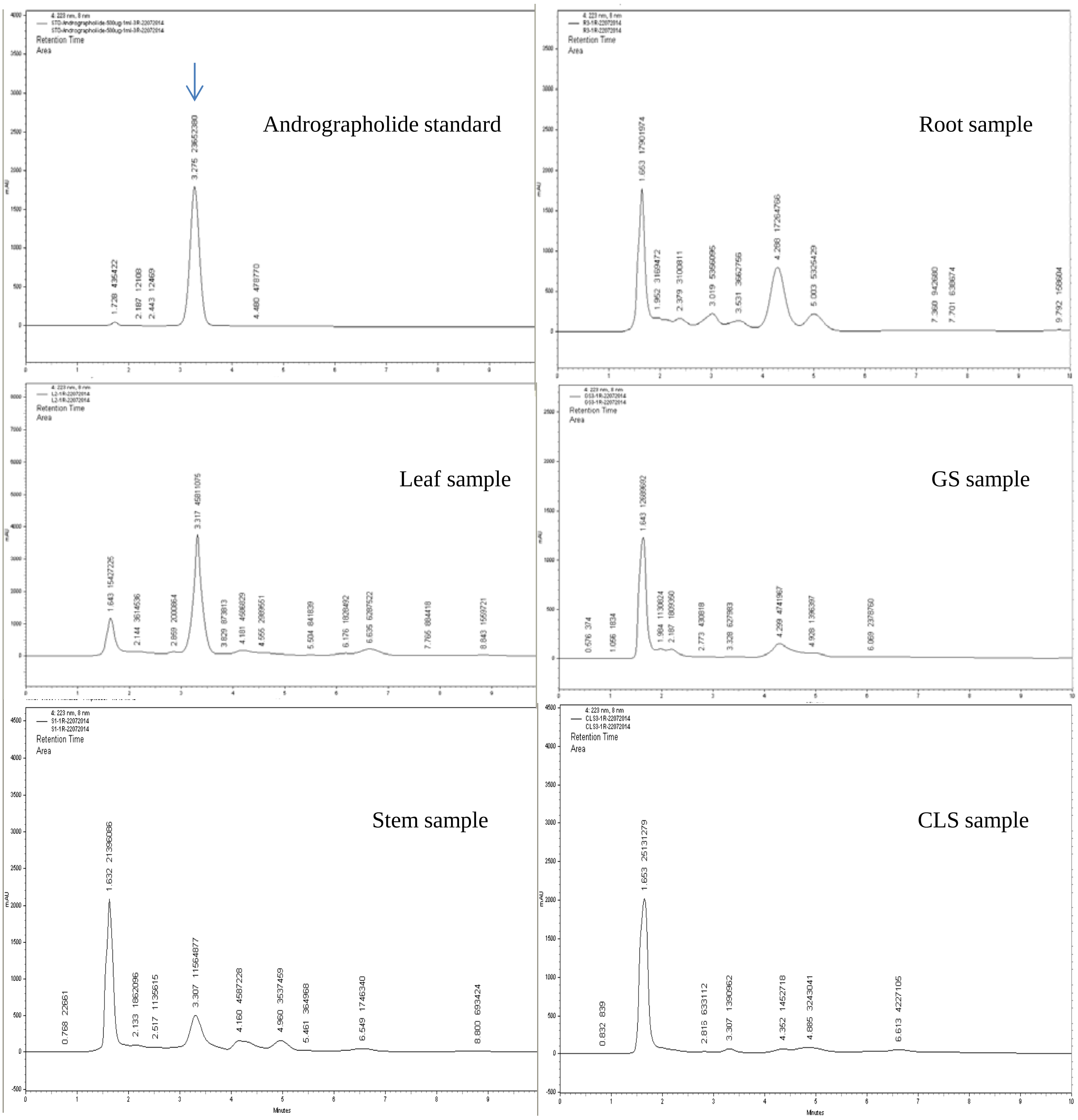

Supplementary Figure S6. HPLC chromatograms of metabolites extracted from anderographolide-accumulating (Leaf and stem) and non-accumulating (root, GS, CLS) tissues of *A. paniculata*. Methanol extracts were analyzed as described in Methods.
