# Supplementary material for: Andrographis paniculata transcriptome provides molecular insights into tissue-specific accumulation of medicinal diterpenes
Source: BMC Genomics. 2015 Sep 2;16(1):659. doi: 10.1186/s12864-015-1864-y (PMC4557604; doi:10.1186/s12864-015-1864-y)
Supplement: Additional file 2: Table S1. — List of the primers used in this study. (DOCX 20 kb) [file 12864_2015_1864_MOESM2_ESM.docx]

**Supplementary Table S1.** List of the primers used in this study.

| **Sl No** | **Target transcripts** |  | **Primer sequences (5′ to 3′)** |
| --- | --- | --- | --- |
|  | ApU55291 (*ApCPS1*) |  | Quantitative RT-PCR |
|  |  | F | GAGGCCGCAACAGTCACAGT |
|  |  | R | GTTGGAGACAGGCGGAGATG |
|  | ApU48901 (*ApCPS2*) | F | CAGAGTCGGTGTGCAGCAA |
|  |  | R | CCAACTCCTCCATCTCCGATT |
|  | ApU53774 (*ApCPS3*) | F | CGCAGAAATCTTCTCAATTTACGA |
|  |  | R | AAGGTTCCATGTCTTCAATGCAGCG |
| 1. 11 | ApU5617 | F | CGTATCCGAGGATCGCAACT |
|  |  | R | AAGGAACGCCGCTTGAGAT |
| 1. 12 | ApU7163 | F | CGGGAAGAGGCGAGGTAGTC |
|  |  | R | CCTCAGGCGGTTTAACAAGAAC |
| 1. 13 | ApU8165 | F | TCCCTCTCGCTCACAAGCA |
|  |  | R | GCCTTGAATGCACTGAAATATGG |
| 1. 14 | ApU8378 | F | CCCTTCACTGTCCAAGTCCACTA |
|  |  | R | AGCTGGCACTGGCAATCG |
| 1. 15 | ApU45495 | F | GGAGCACCGCCGACATAT |
|  |  | R | TGCCATCATGGGTTGCATT |
| 1. 16 | ApU9344 | F | CGTCCGCCTTTCTCAAGATC |
|  |  | R | TCCTGATGAAGTAAACAACGTCAAA |
| 1. 17 | ApU12883 | F | CCATTCCAAGTCCAGAAGATATGC |
|  |  | R | GAAAGCCGGAAGATCTATCCAA |
| 1. 18 | ApU13057 | F | ACATGCCTTCCTACCCAATTACAG |
|  |  | R | GCACATTTGTGGGACGGTTT |
| 1. 19 | ApU67412 | F | TTGACCTAGAATGGCGATTTCTC |
|  |  | R | GGTATCCGGCAAGGAAAGCT |
| 1. 20 | ApU70472 | F | ATCCAGAGTTCTCACCACAAAGG |
|  |  | R | GCTGCTGTTCTCGGAGTTCCT |
| 1. 21 | ApU3039 | F | GTGGGCAGCAATGCTCCTAT |
|  |  | R | GGCAGATCCTTCTGTTGTAAACCT |
| 1. 22 | ApU952 | F | GCCAAAGACTGTGTGGTTGGA |
|  |  | R | CCACGACGACTTGCCATACA |
| 1. 23 | ApU55421 | F | TGGATTTACCGACTTCCAGTACCT |
|  |  | R | CGCTGTGTGCCTTTATTTGTTG |
| 1. 24 | ApU70437 | F | CATTTGCATTGTCTCACCAGTAACT |
|  |  | R | TGGCACTGGCCTCATTAAAAA |
| 1. 25 | ApU80862 | F | ATCCCGCACAATAAAAAGCAGAT |
|  |  | R | TTGGGTCGCATCCTTTATAAGG |
| 1. 26 | ApU50057 | F | GGCCGGAGCGAATTTATTG |
|  |  | R | CGAGCCAAGCTTGCAAAGA |
| 1. 28 | *ApU54408* | F | TAGCCCACGTCGTCGTCAT |
|  |  | R | CGGGACACCAAGTGCAACTT |
| 1. 29 | *ApU45802* | F | GGGAATGGTCGAGTCTGCAT |
|  |  | R | TCAGCTACGAGAAGGCGATATG |
| 1. 30 | *ApU2567* | F | GCCGTCGCTAGAGGAAATCA |
|  |  | R | TGCAAACTCGCTGCGTAGTC |
| 1. 31 | *ApU393* | F | GGGCTGTCCAGATTGCTTATG |
|  |  | F | TCCATCTCTTCGAGCCTCTGA |
|  | *ApU5178* | F | CTCCCGATGGCATTGCTTT |
|  |  | R | AGAGGGTTCGTCCTTCTTGCT |
|  | *ApU61702* | F | CGACACCGAGGTCAAAGCAT |
|  |  | R | TGTTGGGCTCTCGCCAAT |
|  | *ApU17395* | F | CGCCACCGTTCTGATCATAAG |
|  |  | R | GGTGGTTGTTCCGGACATG |
|  | *ApU55028* | F | CCCGAAGCTCACGTAGATGAC |
|  |  | R | GCCCTCTACACGCCCATTTA |
|  | *ApU63394* | F | GGAGGATGATGCACTCGTTGA |
|  |  | R | GGTCACCTTGAAAGCCACCAT |
|  | *ApU52491* | F | TTGGCGAGTCACCATGCA |
|  |  | R | CCATGCTTGGTCCCTCACAT |
|  | ApU2396 | F | GGCGAGCAATGCGAAAAGT |
|  |  | R | CAGCTGCCTTCCCATTCTTG |
|  | *ApU74674* | F | CGCTAACATCGTCCACAGCAT |
|  |  | R | GCGTCGAGGCAGTGATGAA |
|  | *ApU70870* | F | GCACACCGATTGGCAGTCT |
|  |  | R | CGGGTGCTCTTTTTCCAAATAG |
|  | *ApU48529* | F | CGACGAGGCATGTGTGAAGA |
|  |  | R | GGTTGTCGCTGATCACGTAGTC |
|  | *ApU55232* | F | CGCTCATCTCCGCCTTGTT |
|  |  | R | CGCCAGCCAAGCCCTATATA |
|  | *ApU53118* | F | CGCGGACAGGATACTCGAAA |
|  |  | R | TCTGCGAAGTCCTCGTGTTG |
|  | *ApU55817* | F | CGCATCGGAGGAAGTCTTTG |
|  |  | R | ACGTTCGTGTGAGCCTTTGTC |
|  | *ApU8377* | F | CGACAAACAAGGGTGGCTACA |
|  |  | R | AGGCGATCCACGATGAAGAG |
|  | *ApU47805* | F | GGAAGATACGCAGCCGTGTT |
|  |  | R | TCAACCCCGGGAGATTTTC |
|  | *ApU46382* | F | CATGGAAGTGATCGGCTTATCC |
|  |  | R | GGCTTCGCACACCACTGATT |
|  | ApU57524 | F | GACATTCGCTGCGGGTCTAG |
|  |  | R | CCCGGTCCATGATGAACCT |
| 1. 32 | *Actin* | F | ACGATGTTCACGGGCATTG |
|  |  | R | GAGCCACCACCTTGATCTTCA |
