# Supplementary material for: Andrographis paniculata transcriptome provides molecular insights into tissue-specific accumulation of medicinal diterpenes
Source: BMC Genomics. 2015 Sep 2;16(1):659. doi: 10.1186/s12864-015-1864-y (PMC4557604; doi:10.1186/s12864-015-1864-y)
Supplement: Additional file 10: Table S9. — A summary of SSRs identified in leaf and root transcriptomes. (DOCX 11 kb) [file 12864_2015_1864_MOESM10_ESM.docx]

**Table S9.** A summary of SSRs identified in leaf and root transcriptomes.

| **Description** | **Leaf** | **Root** |
| --- | --- | --- |
| Total number of sequences examined | 69011 | 64244 |
| Total size of examined sequences (bp) | 46032575 | 44489873 |
| Total number of identified SSRs | 16485 | 15911 |
| Number of SSR containing sequences | 13805 | 13213 |
| Number of sequences containing more than one SSR | 2194 | 2200 |
| Number of compound SSRs | 1877 | 1895 |
| Di-nucleotide (≥6 repetitions) | 5194 | 5023 |
| Tri-nucleotide (≥5 repetitions) | 3984 | 4047 |
| Tetra-nucleotide (≥5 repetitions) | 528 | 508 |
| Penta-nucleotide (≥5 repetitions) | 138 | 137 |
| Hexa-nucleotide (≥5 repetitions) | 125 | 114 |
